# Exploring the 2′-Hydroxy-Chalcone Framework for the Development of Dual Antioxidant and Soybean Lipoxygenase Inhibitory Agents

**DOI:** 10.3390/molecules26092777

**Published:** 2021-05-08

**Authors:** Ioanna Kostopoulou, Andromachi Tzani, Nestor-Ioannis Polyzos, Maria-Anna Karadendrou, Eftichia Kritsi, Eleni Pontiki, Thalia Liargkova, Dimitra Hadjipavlou-Litina, Panagiotis Zoumpoulakis, Anastasia Detsi

**Affiliations:** 1 Laboratory of Organic Chemistry, Department of Chemical Sciences, School of Chemical Engineering, National Technical University of Athens, Heroon Polytechniou 9, Zografou Campus, 15780 Athens, Greece; ioanna.th.kostopoulou@gmail.com (I.K.); andromachi.tzani@gmail.com (A.T.); nestor_polyzos@hotmail.com (N.-I.P.); markarad96@gmail.com (M.-A.K.); 2 Institute of Chemical Biology, National Hellenic Research Foundation, 48, Vas. Constantinou Avenue, 11635 Athens, Greece; ekritsi@eie.gr (E.K.); pzoump@uniwa.gr (P.Z.); 3 Department of Food Science and Technology, University of West Attica, Ag. Spyridonos, 12243 Egaleo, Greece; 4 Laboratory of Pharmaceutical Chemistry, School of Pharmacy, Faculty of Health Sciences, Aristotle University of Thessaloniki, 54124 Thessaloniki, Greece; epontiki@pharm.auth.gr (E.P.); thalialiargkova@yahoo.gr (T.L.); hadjipav@pharm.auth.gr (D.H.-L.)

**Keywords:** chalcones, aurones, butein, sulfuretin, antioxidant activity, lipoxygenase inhibition, molecular docking

## Abstract

2′-hydroxy-chalcones are naturally occurring compounds with a wide array of bioactivity. In an effort to delineate the structural features that favor antioxidant and lipoxygenase (LOX) inhibitory activity, the design, synthesis, and bioactivity profile of a series of 2′-hydroxy-chalcones bearing diverse substituents on rings A and B, are presented. Among all the synthesized derivatives, chalcone **4b**, bearing two hydroxyl substituents on ring B, was found to possess the best combined activity (82.4% DPPH radical scavenging ability, 82.3% inhibition of lipid peroxidation, and satisfactory LOX inhibition value (IC_50_ = 70 μM). Chalcone **3c**, possessing a methoxymethylene substituent on ring A, and three methoxy groups on ring B, exhibited the most promising LOX inhibitory activity (IC_50_ = 45 μM). A combination of in silico techniques were utilized in an effort to explore the crucial binding characteristics of the most active compound **3c** and its analogue **3b**, to LOX. A common H-bond interaction pattern, orienting the hydroxyl and carbonyl groups of the aromatic ring A towards Asp768 and Asn128, respectively, was observed. Regarding the analogue **3c**, the bulky (-OMOM) group does not seem to participate in a direct binding, but it induces an orientation capable to form H-bonds between the methoxy groups of the aromatic ring B with Trp130 and Gly247.

## 1. Introduction

**Figure 1:**
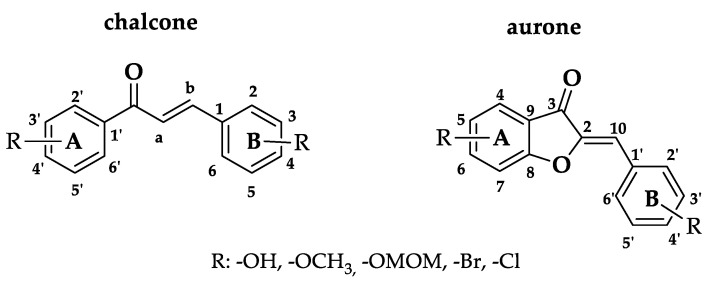
General structure and numbering scheme of the chalcone and aurone framework.

Chalcones (1,3-diaryl-2-propen-1-ones) (Figure 1) constitute a large and important group of bioactive compounds belonging to the flavonoid family. They possess an important role in medicinal chemistry since they are privileged scaffolds used as lead compounds for the discovery of new drugs. Thus, the isolation from natural sources (plants, flowers, fruits, vegetables etc.), as well as the organic synthesis of chalcones have been intensified during the last decades due to their wide spectrum of bioactivity. The presence of a α,β-unsaturated carbonyl system in chalcone derivatives enable them to exhibit a wide range of biological activities such as antioxidant, anti-inflammatory, [1], anticancer [2], antibacterial [3], antimalarial [4], tyrosinase inhibitory activity [5], anti-HIV [6], anti-leishmanial [7], while they are also potential neuroprotective agents [8,9]. The chalcone derivatives which possess hydroxyl and methoxy substitution on aromatic rings A and B have demonstrated potential anti-leishmanial and anti-trypanosomal activities by acting on a number of molecular targets. Moreover, free hydroxyl groups are essential to increase their activity (such as antioxidant and antimicrobial) while methoxy groups decrease it. Regarding antitumor activity, it is possibly positively affected in the presence of methoxy substitutions on the aromatic ring [10].

Another flavonoid subclass, aurones ((Z)-2-benzylidenebenzofuran-3-(2H)-ones) (Figure 1) are oxidative cyclization products of chalcones that rarely appear in nature and are less studied than chalcones. So far aurones have been exploited via isolation from flowering plants [11,12], invasive weeds [13], mosses [14], and marine brown algae [15], via organic synthesis [16,17] and biosynthesis from chalcone precursors by the key enzyme aureusidin synthase [18]. Sulfuretin [19] and aureusidin [20] consist representative naturally occurring aurones, bearing different hydroxylation patterns, while some methoxy-aurones also occur in nature [21,22]. Aurones constitute promising bioactive compounds exhibiting among others, antioxidant [17], antifungal [23], anticancer [24], antimicrobial [25], anti-leishmanial [16], antimalarial [26], anti-inflammatory activity [27], and consist potential effective agents for the treatment of Alzheimer’s disease [28]. Consequently, during the last decades, there is an increase in publications concerning their isolation, synthesis, and bioactivity evaluation.

In this work, among the synthesized compounds the synthesis of two naturally occurring chalcones and aurones, such as butein (**4a**) and sulfuretin (**7a**), is also presented.

Butein (2′,4′,3,4-tetrahydroxy-chalcone) occurs in many unrelated genera including *Butea Dahlia*, *Coreopsis*, and *Searsia* and has been isolated in good yields [29]. Chemically, butein has been synthesized through various organic synthetic methods including microwave irradiation in the presence of PdCl_2_(PPh_3_)_2_, ammonium formate in THF [30], aldol condensation catalyzed by the novel acid catalyst thionyl chloride (SOCl_2_)/ethyl alcohol (EtOH) [31], Claisen–Schmidt condensation between protected 2,4-dihydroxyacetophenone and 3,4-dihydroxybenzaldehyde in the presence of basic catalyst, and subsequent removal of the protecting groups [30,32].

Among naturally occurring aurones, sulfuretin (6,3′,4′-trihydroxyaurone) is known to have anti-inflammatory effects and has been isolated among others from the heartwood of *Rhus verniciflua*, *Bidens*, *Cosmos*, and *Dahlia* flowers [33,34]. Furthermore, the total synthesis of sulfuretin has been exploited by scientists starting by the chemical synthesis of a 3-coumaranone, obtained by gold(I)-catalyzed oxidative cyclization of an o-ethynylanisole and its condensation with 3,4-dimethoxybenzaldehyde in basic conditions, followed by demethylation with BBr_3_ [35]. To our knowledge, the synthesis of sulfuretin has not been previously achieved using the oxidative cyclization of the OMOM protected butein precursor.

Natural and synthetic chalcones have shown to possess strong radical scavenging potential, while some of them, such as 2,3,4,6-tetrahydroxychalcone and butein (2′,4′,3,4-tetrahydroxy-chalcone), exhibit more potent radical-scavenging activity than reference antioxidants such as vitamin C or α-tocopherol [36,37]. It is considered that hydroxyl groups consist key moieties which can enhance the antioxidant activity of chalcones [23,38]. Aurones have been less studied than chalcones concerning their antioxidant and anti-inflammatory activity whereas their ability to inhibit soybean lipoxygenase (LOX) and histone deacetylase (HDAC) have been recently reported [27,39,40,41].

The combined anti-inflammatory and antioxidant activity of novel drugs could be an asset for the treatment of several diseases and several natural and synthetic antioxidants have been shown to possess potent anti-inflammatory properties [42,43]. In this framework, and as a continuation of our studies towards the synthesis and biological evaluation of novel bioactive compounds [16,17,44,45], we present herein the synthesis and structure identification of synthetic and naturally occurring 2′-hydroxy-chalcones and selected aurones. Among the 2′-hydroxy-chalcones series (**3a**–**3l**, **4b**, and **5**) and aurones (**6a**–**6b** and **7a**–**7b**), the chalcones **3c**, **3h**, **4b**, **5** and the aurone **6a** are presented, to our knowledge, for the first time in the literature. The novel chalcones were prepared via Claisen–Schmidt condensation in alkaline media (Method A: 20% *w/v* KOH, EtOH, Method B: 60% *w/w* NaH, DMF). Chalcones **3f**–**3g** and **3i**–**3l**, were prepared through the Claisen–Schmidt condensation reaction between appropriately substituted 2′-hydroxy-acetophenones and benzaldehydes carried out using aqueous KOH in ethanol, as previously reported in the literature [41,46,47,48,49]. Aurones **6b** and **7b** were synthesized via oxidative cyclization of the corresponding chalcones using mercury (II) acetate in pyridine followed by deprotection of the methoxylated aurone using BBr_3_ in CH_2_Cl_2_, respectively. The synthesis of aurones **6b** and **7b** has been previously presented [50,51] via Knoevenagel condensation of benzofuranone with the appropriate benzaldehyde using polyphosphoric acid (PPA).

The synthesized compounds were evaluated in vitro for their antioxidant activity as well as their ability to inhibit soybean LOX. Structure/activity relationship and in silico studies were undertaken, attempting to discover the structural characteristics of compounds **3b** and **3c** that contribute to their LOX inhibitory activity.

## 2. Results and Dicussion

### 2.1. Chemistry

First, 2-Hydroxy-4-(methoxymethoxy)-acetophenone **1a** and 3,4-bis(methoxymethoxy)-benzaldehyde **2a** were synthesized from the corresponding hydroxy-compounds using MOM-Cl and K_2_CO_3_ in acetone. Further, 2′-hydroxy-acetophenones **1b**–**1e** and benzaldehydes **2b**–**2h** were commercially available.

**Scheme 1:**
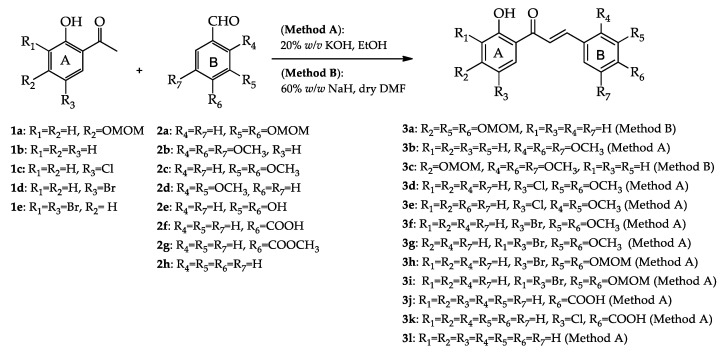
Synthetic procedure of 2′-hydroxychalcones **3a**–**3l**.

Chalcones **3a**–**3l** were obtained in 22–85% yields via Claisen–Schmidt condensation of 2-hydroxy-acetophenones **1a**–**1e** with appropriately substituted benzaldehydes **2a**–**2h** in basic conditions (Scheme 1).

**Scheme 2:**
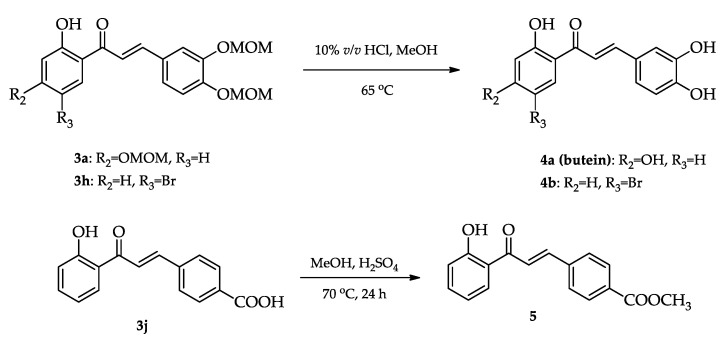
Synthetic procedure of 2′-hydroxychalcones **4a**, **4b**, and **5**.

The hydroxylated chalcones **4a** and **4b** were prepared by deprotection of the corresponding chalcones **3a** and **3h**, respectively, after removal of the methoxymethyl-protecting group, using 10% aqueous HCl in methanol. Chalcone **5** was synthesized by Fischer esterification of the corresponding chalcone **3k** in methanol, using H_2_SO_4_ as a catalyst (Scheme 2).

**Scheme 3:**
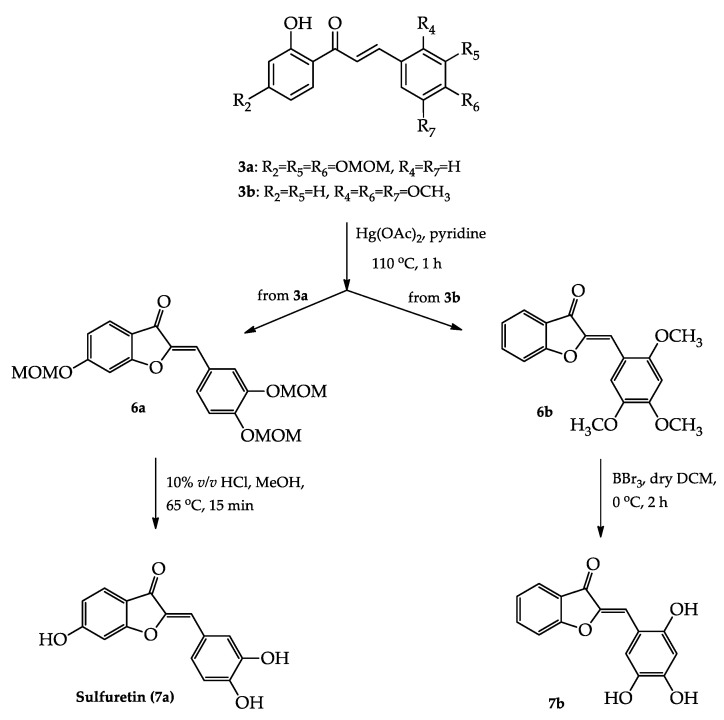
Synthetic procedure of aurones **6a**, **6b**, sulfuretin (**7a**) and **7b**.

Furthermore, aurones **6a** and **6b** were obtained in moderate yields (60 and 74% respectively) via the oxidative cyclization of the corresponding chalcones **3a** and **3b**, using mercury(II) acetate in pyridine as has been described in detail in one of our previous publications [17]. Aurone **7a** was prepared by deprotection of the corresponding aurone **6a**, by removing the methoxymethyl-protecting group using 10% aqueous HCl in MeOH, whereas aurone **7b** was prepared by deprotection of the methoxylated aurone **6b** using BBr_3_ in CH_2_Cl_2_ as the deprotecting agent (Scheme 3).

The structural characterization of the synthesized compounds was confirmed by ^1^H- and ^13^C-NMR, MS/ESI (+/−), and HRMS spectroscopy (for those compounds that have not been reported in the literature).

The ^1^H-NMR spectra of 2′-hydroxy-chalcones are characterized by a signal at a low field (≈13 ppm) attributed to the proton of the 2′-OH group which is deshielded as a result of taking part in a strong intramolecular hydrogen bond with the neighboring carbonyl group, and the pair of AB doublets with characteristic *J* values of 15.0–17.0 Hz, confirming the E-geometry of the double bond. In the ^1^H-NMR spectra of chalcones possessing methoxy group on position 2 (chalcones **3b**, **3c**, **3e**), a significant downfield shift of the AB doublets is observed for the vinyl protons which appear at 8.01–8.21 ppm (for Hb) and 7.54–7.92 ppm (for Ha) as compared to the same signals in the spectra of the chalcones (**3a**–**3d**, **3f**–**3i**, **3k**, **3l**, **4a**, **4b**) without substitution at position 2 (7.68–8.05 ppm for Hb and 7.45–7.87 ppm for Ha, respectively). This can be attributed to the ‘ortho’ effect of the methoxy group. In the ^1^H-NMR spectra of the synthesized aurones, the most characteristic peak is the singlet owed to the exocyclic vinylic proton at δ 6.78–7.50 ppm for the substituted on ring A. The presence of a substituent on position 2′ in aurones **6b** and **7b** causes a downfield shift of the vinylic proton signal at 7.78 ppm and 7.74 ppm, respectively. This difference in chemical shifts is also attributed to the ‘ortho’ effect [17].

### 2.2. In Silico Determination of Lipophilicity as ClogP

Lipophilicity is an important physicochemical property related to the biological activity and ADME properties. We theoretically calculated the lipophilicity values of all compounds as ClogP using the CLOGP Program of Biobyte Corp. [52].

### 2.3. Bioactivity Assessment

In this work, we evaluated in vitro the bioactivity of a series of chalcones and aurones that were expected to offer protection against inflammation and free radical attack.

Free radicals, which are generated during the biochemical function of all aerobic organisms, are very reactive species that can attack and damage biological targets such as lipids and DNA. Normally, the endogenous antioxidant mechanisms of the organisms (redox enzymes and small molecules such as vitamins) scavenge the free radicals, however these mechanisms are not effective enough when excessive free radical production occurs. In this case, exogenous antioxidants have to be used in order to prevent damage caused by oxidative stress. Antioxidants are defined as substances that even at low concentration, significantly delay or prevent oxidation of easily oxidizable substrates [53,54].

There is an increased interest of using antioxidants for medicinal purposes in the recent years, as it is widely accepted that free radicals play a crucial role in many pathological conditions such as inflammation, cancer, and neurodegenerative disorders [55,56,57,58]. Moreover, several nonsteroidal anti-inflammatory agents have been reported to act either as inhibitors of free radical production or as radical scavengers [59]. Consequently, compounds possessing antioxidant properties could be expected to offer protection in inflammation and to lead to potentially effective drugs.

In order to estimate the antioxidant activity of a compound, different experimental approaches are used. In this way, factors such as solubility or steric hindrance, which may be of overriding importance in one environment but not in another, can be varied and the antioxidant ability of a compound in a variety of milieus may be evaluated. Most of them require a spectrophotometric measurement and a certain reaction time in order to obtain reproducible results [60].

In this context, the in vitro antioxidant capacity of the synthesized chalcones and aurones (Table 1) was monitored in terms of: (a) the interaction with the stable free radical 2,2-Diphenyl-1-picrylhydrazyl (DPPH); (b) the anti-lipid peroxidation activity; (c) the hydroxyl radical scavenging ability; and (d) the 2,2-azino-bis(3-ethylbenzothiazoline-6-sulfonic acid (ABTS) radical cation (ABTS^•+^) reduction-decolorization ability.

The first assay refers to the interaction of the compounds with the stable free radical DPPH and it is a fast, simple, cost-effective, and widely used method, where the DPPH radical reacts directly with the antioxidant and is decolorized. This interaction indicates their radical scavenging ability in an iron-free system. The scavenging effect of the synthesized analogues on the DPPH radical was evaluated according to the methods of Hadjipavlou et al. [61].

The second technique is based on the ability of the compounds to inhibit lipid peroxidation of linoleic acid induced by 2,2′-Azobis(2-amidinopropane) dihydrochloride (AAPH) radical (Table 2). AAPH-induced linoleic acid oxidation has been developed as a quick and reliable method for measuring the antioxidant activity. The thermal free radical producer (AAPH) generates free radicals in the solution which cause the oxidation of linoleic acid, and the method is a measure of how effective antioxidants protect against lipid peroxidation in vitro. Oxidation of linoleic acid by AAPH is followed by UV spectrophotometry in a highly diluted sample [61].

Among the reactive oxygen species (ROS), the hydroxyl (^•^OH) free radical is possibly the most toxic, reacting with several biological important molecules such as DNA, lipids, or carbohydrates. Polyunsaturated fatty acids are particularly vulnerable by free radicals. Iron released from damaged cells is more likely to be readily available to catalyze the generation of ^•^OH radicals. Thus, we tried to test the ability of our compounds to scavenge hydroxyl radicals determining the competition of selected chalcones and aurones with DMSO for hydroxyl radicals. Hydroxyl radicals were generated using the Fe^3+^/ascorbic acid system and expressed as a percentage inhibition of formaldehyde production in the presence of each compound at 100 μM.

The ABTS radical cation decolorization assay was used as another antioxidant activity estimation protocol (Table 2). Oxidation of ABTS with potassium persulfate generates directly the radical with no involvement of an intermediate radical, which is then reduced upon adding electron-donating antioxidants.

Furthermore, as an indication of their anti-inflammatory activity, all the molecules were tested as inhibitors of soybean lipoxygenase (LOX). The enzyme lipoxygenase catalyzes the first two steps in the metabolism of arachidonic acid, which is cleaved from membrane phospholipids to leukotrienes (LTB4). LTB4 generation is considered to be important in the pathogenesis of neutrophil-mediated inflammatory diseases. For this study, the UV absorbance-based soybean LOX assay was used, which is a plant enzyme with satisfactory homology with the human 5-LOX [61,62,63,64,65,66].

#### 2.3.1. Antioxidant Activity

The results of the DPPH scavenging ability of all the synthesized compounds (Table 2) revealed that, for the active analogues, the result was not time dependent (from 20 to 60 min). In the DPPH assay, the basic chemical reaction involved is the reduction of the DPPH radical by a single electron transfer (ET) from the antioxidant. Particularly effective antioxidants via this mechanism are the phenoxide anions derived from phenolic compounds, like catechol and derivatives, such as Nordihydroguaiaretic acid (NDGA). Chalcone analogues **3a** and **3f**–**3i**, which possess -OCH_3_ or -OMOM substituents at ring B, seem either to be totally inactive (**3f**, **3g**, **3i**) in scavenging the DPPH radical, or to possess weak activity (**3a**, **3h**); whereas their hydroxylated analogues **4a** and **4b** exhibit significant interaction with the radical showing DPPH radical scavenging ability (95.7% and 82.4%, respectively).

The evaluation of the antioxidant activity of aurone derivatives via the DPPH method shows that aurones **6a** and **6b** exhibit no interaction with the radical, whereas their hydroxylated analogues, sulfuretin (**7a**) and aurone **7b**, are potent DPPH scavengers with 89% and 100% inhibition, respectively. NDGA was used as a reference compound (Table 2).

All the synthesized chalcones possess a phenolic hydroxyl group at position 2′ of the aromatic ring A. However, this OH cannot effectively interact with the DPPH radical as it is involved in a strong hydrogen bond with the adjacent carbonyl oxygen. The antioxidant activity of 2′-hydroxy-chalcones arises mainly from the other substituents on rings A and B, as was also observed in our previous work regarding quinolinone-chalcone hybrids [67].

Furthermore, it seems that the presence of bromo-substituent in chalcone derivatives, in combination with the presence of hydroxyl groups, enhances the antioxidant activity of the compounds. The chalcones **4a** and **4b** exhibit high antioxidant activity regarding the DPPH assay (95.7% and 82.0%, respectively), while the **4b** derivative, which possesses one bromo-substituent at position 5′ of the aromatic ring A, shows significantly higher lipid peroxidation inhibitory activity (82.3%), than the **4a** analogue, which is almost inactive (24.4%). The insertion of the Br substituent renders the chalcone **4b** more lipophilic (ClogP 3.14) than chalcone **4a** (ClogP 1.65) and this can possibly explain the enhanced lipid peroxidation inhibitory activity of **4b**. Aurones **6a** and **6b** did not show any DPPH radical scavenging ability, exactly as their chalcone precursors **3a** and **3b**.

Sulfuretin (**7a**) showed potent DPPH scavenging activity (89.0/91.2%) while the 2,4,5-trihydroxy aurone **7b** was one of the most active compounds in this assay, showing 100% inhibition.

Lipophilicity as ClogP values does not seem to play any significant role in the DPPH scavenging ability of the compounds (Table 5).

As far as the lipid peroxidation inhibitory ability of the tested chalcones is concerned, it seems that the presence of bromo-substituents enhances anti-lipid peroxidation. Chalcone **3h**, which bears two -OMOM substituents at positions 3 and 4 of ring B and 3,4-dihydroxychalcone derivative **4b**, which both possess a bromo-substituent at position 5′, exhibit satisfactory inhibitory activity of 65.6% and 82.3%, respectively. The introduction of one more bromo-substituent at position 3′, leads to compound **3i**, which is strong inhibitor of lipid peroxidation (93%). However, chalcones **3f** and **3g**, which also possess bromo-substituents, are inactive against lipid peroxidation. This is maybe attributed to the existence of two methoxy groups at the aromatic ring B, which seem to lead to inactive inhibitors, as it also happens in the case of chalcones **3d** and **3e**. Furthermore, according to Table 2, it seems that the presence of a chloro-substituent at the aromatic ring A of chalcone moiety leads to inactive molecules. Among all the evaluated chalcones, compound **5** with a methoxy-carbonyl substituent at aromatic ring B showed the best inhibitory activity (100%). The studied aurones exhibited low activity overall, with the exception of aurone **7b** which showed a moderate lipid peroxidation inhibition of 53.2%. With some exceptions anti-lipid peroxidation goes in parallel to lipophilicity values.

The % values for DPPH scavenging activity and AAPH anti-lipid peroxidation activity in relation to time, for the most potent compounds are given in Table 3 and Table 4. Both activities seem to be constantly high during the 10 min of observation and measurement. These results verify the strong antioxidant capacity of the tested compounds, which is exhibited immediately after their addition to the assay medium (t = 0 min) and rapidly reaches a plateau at t = 9 min.

Representative compounds were selected for the competition with DMSO for hydroxyl radicals assay (Table 2). The best activity was shown by chalcone **4b** whereas aurone **7b**, with a 2′,4′,5′-trihydroxy-substitution pattern on ring B, follows. The presence of a hydroxyl substituent at ring A of the aurone scaffold seems to affect this type of activity: the natural product sulfuretin (**7a**), with a 5-OH at ring A and a catecholic moiety at ring B was inactive in this assay. Chalcone **4a**, the precursor of sulfuretin (**7a**) shows 68% competition with DMSO for hydroxyl radicals. The highly oxygenated aurone **6a** presents moderate activity, equal to its precursor, chalcone **3a** (43 and 40%, respectively). Trolox was used as a reference compound (Table 2).

In the ABTS radical cation (ABTS^•+^) decolorization assay (Table 2), the tested chalcones showed better activity than the corresponding aurones (compare **3a** with **6a** and **4a** with **7a**), with the exception of the 2′,4′,5′-trimethoxy chalcone **3b** and chalcone **4b**, which were both inactive, whereas aurone **6b** showed 64% activity. Trolox was used as a reference compound (Table 2).

#### 2.3.2. Soybean LOX Inhibitory Activity

In the present series of compounds, chalcones were generally found to possess significantly higher LOX inhibitory activity than aurones (Table 5). This is in accordance with our previous study [17].

Among all the tested chalcones, the most potent lipoxygenase inhibitor is chalcone **3c** (IC_50_ = 45 μM) which possesses one -OMOM group at position 4′ of ring A and three methoxy groups at positions 2, 4, and 5 at ring B. The -OMOM groups seems to play an important role in the LOX inhibitory activity of this series of 2′-hydroxy-chalcones: Chalcone **3b**, which possesses the same methoxy substitution pattern as chalcone **3c** but lacks the -OMOM group at ring A, shows lower activity (IC_50_ = 75 μM) than **3c**. Chalcone **3a**, with a 4′-OMOM and 3,4-di-OMOM substitution is a poor LOX inhibitor (40.2%). The presence of two -OMOM substituents at positions 3 and 4 of ring B favors LOX inhibitory activity in the case of chalcones **3h** and **3i**, which also bear bromine substituents at ring A. In fact, chalcone **3h** with a 5′-Br substitution pattern is the second most active LOX inhibitor of the series (IC_50_ = 55 μM) whereas **3i**, with an additional Br atom at position 3′, shows IC_50_ = 67.5 μM. NDGA was used as a reference compound (Table 5).

The -OMOM group seems also to be a structural feature that affects activity depending on the substituents at ring A: Chalcone **3i**, with 3′, 5′-dibromo pattern at ring A and 3,4-di-OMOM at ring B, is almost equipotent with its 3,4-dimethoxy analogue **3g** (IC_50_ = 67.5 and 68.5 μM, respectively). However, chalcone **3h**, one of the most active of the series with one bromine substituent at position 5′, is transformed to an inactive compound when the -OMOM groups are replaced by -OCH_3_ (chalcone **3f**, 38% inhibition). On the other hand, removal of the -MOM protecting group results to the active 3,4-dihydroxy chalcone analogue **4b** (IC_50_ = 70 μM).

Chalcone **3d**, the chlorine-containing analogue of **3f**, is also inactive but a change of the position of the methoxy groups at ring B results to a significant improvement in activity: chalcone **3e** with a 2,3-dimethoxy substitution pattern shows IC_50_ = 100 μM.

The transformation of chalcone **3b** to the analogous aurone **6b** diminishes the activity, showing only 13.0% LOX inhibition. The same decrease in inhibition is observed in the transformation of **4a** to **7a** and of **3a** to **6a**.

As a general remark, lower inhibition results are observed by the compounds that possess lower lipophilicity values.

### 2.4. In Silico Studies

#### 2.4.1. Molecular Docking Studies

The lack of a co-crystallized ligand in the crystal structure of soybean lipoxygenase isoform 1 (LOX-1) (PDB ID: 3PZW) imposes the identification of potential allosteric binding sites apart from the iron-binding site and the substrate-binding cavity. To this extent, three putative binding sites were identified using SiteMap’s approach [68,69,70]. In particular, Site 1 is located and mapped at the interface of the amino terminal β-barrel (PLAT domain) and of the α-helical domain (houses the catalytic iron) while Sites 2 and 3 belong to the α-helical domain (Figure S1, Supplementary Materials) [71]. Evaluation of Site Score, indicate Site 1 (site score 1.113) as a putative allosteric site for further docking studies. Interestingly, Site 1 has been previously identified and utilized for docking studies on LOX-1, as presented in recent publications [67,72].

Molecular docking was utilized to study the binding mode of the most promising synthesized chalcone **3c** and its analogue **3b**, which does not possess an -OMOM substituent, in an effort to provide a structure-activity explanation on soybean LOX-1 inhibitory activity. Additionally, for comparison reasons, the binding mode of the standard LOX inhibitor NDGA (IC_50_ = 0.45 μΜ) was determined. In general, the results suggested that the studied compounds interact with the soybean LOX-1 through allosteric interactions.

**Figure 2:**
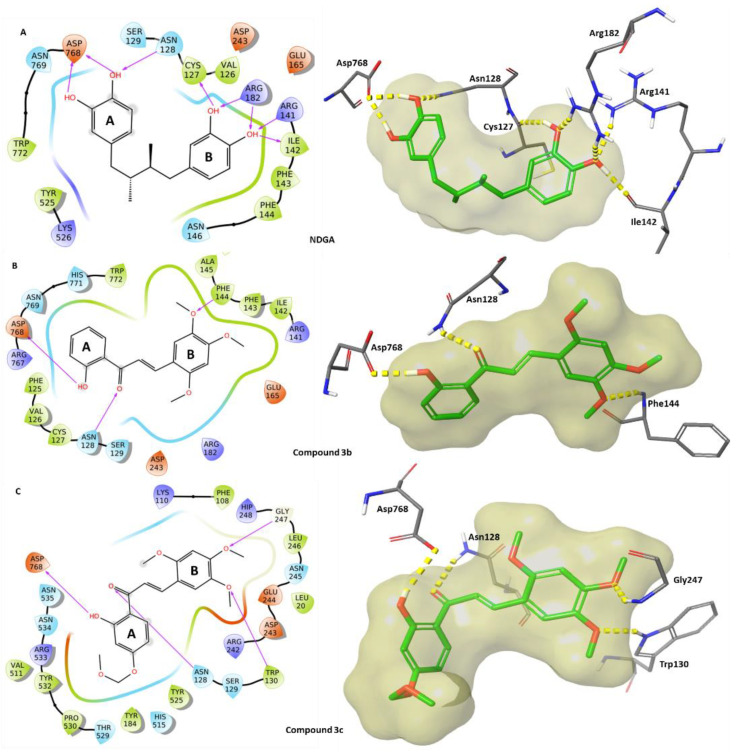
2D and 3D representative binding poses of (**A**) nordihydroguaiaretic acid (**NDGA**), (**B**) Compound **3b**, and (**C**) Compound **3c** are depicted. Hydrogen bonds are illustrated with pink (2D) and yellow (3D) color lines. Figure made with MAESTRO (Schrödinger Release 2020-4: Maestro, Schrödinger, LLC, New York, NY, USA, 2020).

Especially, the analysis of the docked poses of the examined compounds **3b** (IC_50_ = 75 μM) and **3c** (IC_50_ = 45 μM) illustrated a common H-bond interaction pattern as depicted in Figure 2, orienting the hydroxyl (-OH) and carbonyl (-CO) groups of their aromatic ring A towards Asp768 and Asn128, respectively. The binding motif of NDGA includes the formation of H-bonds between the hydroxyl groups (-OH) of the aromatic ring A with Asp768 and Asn128, highlighting their crucial role in the binding and potentially in the inhibitory activity. Additionally, the hydroxyl groups (-OH) of the aromatic ring B of NDGA create a strong hydrogen bond network, including Cys127, Arg182, Arg141, and Ile142, which is not presented in the examined **3b** and **3c**. However, in the case of **3c**, although the bulky (-OMOM) group seems not to participate in a direct binding, it induces an orientation capable to form H-bonds between the methoxy (-OCH_3_) groups in positions 4 and 5 of the aromatic ring B with Trp130 and Gly247. In the case of **3b** only, the methoxy (-OCH_3_) group in position 3 forms a H-bond with Phe144, probably inducing the reduction of the inhibitory activity (Figure 2).

#### 2.4.2. Molecular Dynamics Simulations Studies

**Figure 3:**
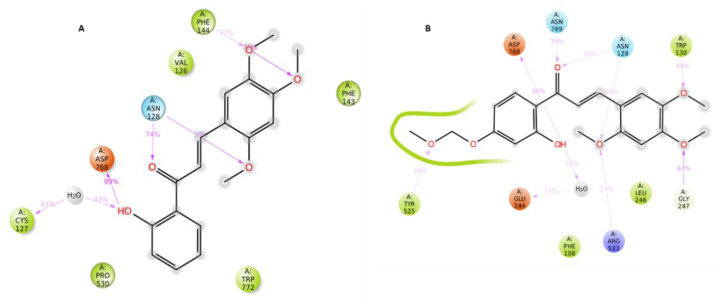
Schematic illustration of LOX-1 interactions throughout the simulation time (10 ns) with (**A**) Compound **3b** and (**B**) Compound **3c**. Interactions that occur more than 20% of the simulation time are presented. Hydrogen bonds are illustrated with pink color lines. Figure made with Desmond software [66].

In a step further, in an effort to evaluate (a) the stability of the tested complexes, (b) the binding mode, and (c) the influence of -OMOM substitution in position 4′ of compound **3c**, short length (10 ns) molecular dynamics simulations were implemented in the docking poses of compounds **3b** and **3c**. LOX-1 interactions as derived from the MD simulations with the testing compounds are depicted in Figure 3. The RMSD results indicated that the structural model backbone of LOX-1, as well as compounds **3b** and **3c**, remain stable during the simulation (Figure S2, Supplementary Materials).

Both compounds exhibit the interactions presented in docking studies (Figure S3, Supplementary Materials). Specifically, the MD results indicate that both **3b** and **3c** preserve the direct H-bonds of their hydroxyl and carbonyl groups with the side chains of Asp768 and Asn128 although not in high percentage (Figure 3). In addition, their methoxy (-OCH_3_) groups in position 2 interact with the backbone (in the case of **3b**) and the side chain (in the case of **3c**) of Asn128, respectively. Moreover, a weaker water-bridged H-bond is formed between the hydroxyl group of compound **3b** and the backbone of Cys127, while the same interaction is shown in the case of compound **3c** with the side chain of Glu244. Regarding compound **3c**, its carbonyl and methoxy (-OCH_3_) group in position 2 interact through H-bonds with the side chains of Asn769 and Arg533, respectively. In contrast, the described interactions are not present for compound **3b**, offering a putative explanation of the higher inhibitory activity of compound **3c**.

Regarding the interactions of methoxy (-OCH_3_) groups at positions 4 and 5 of **3b** and **3c**, a different profile is observed in agreement with the docking results. Especially, for compound **3b**, the methoxy (-OCH_3_) groups share H-bonds with Phe144 backbone while in the case of compound **3c**, H-bonds are formed with Gly246 and Trp130.

With regard to the -OMOM chain of compounds **3c**, the MD simulations indicates the formation of a hydrogen bond with Tyr525.

Overall, the binding of **3b** and **3c** is reinforced by additional hydrophobic interactions. In detail, compound **3b** interacts with Val126, Val520, and Trp772 while compound **3c** develops interactions with Leu246 and Phe108 (Figure S3, Supplementary Materials).

## 3. Materials and Methods

NMR spectroscopy: Synthesized compounds were structurally elucidated using Varian Gemini 300 MHz (Palo Alto, CA, USA) at the School of Chemical Engineering, NTUA and Varian 600 MHz (Palo Alto, CA, USA) at the Institute of Chemical Biology, NHRF, NMR spectrometers using DMSO-d_6_, CDCl_3_ 99.9 atom % D and CD_3_OD-d_4_ as solvents. ^1^H-NMR spectra were recorded at 298 K under the following parameters. Spectral width (SW) = −2–14 ppm, number of scans (ns) = 32. Coupling constants (*J*) are expressed in hertz (Hz). Chemical shifts (δ) of NMR are reported in parts per million (ppm) units relative to the solvent.

Melting points were determined on a Gallenkamp MFB-595 melting point apparatus (London, UK) and are uncorrected. ESI-MS spectra were recorded on a Varian 500 MS IT Mass Spectrometer (Palo Alto, CA, USA). For the in vitro tests, a Lambda 20 (Perkin–Elmer, Norwalk, CT, USA) UV–Vis double beam spectrophotometer was used.

### 3.1. Synthesis and General Procedures

*2-hydroxy-4-(methoxymethoxy)-acetophenone* (**1a**) [17]: To a mixture of 2,4-dihydroxy-acetophenone (531.1 mg, 3.5 mmol) and anhydrous K_2_CO_3_ (1.9 g, 10.0 mmol) in dry acetone (39 mL) was added dropwise chloromethyl methylether (CH_3_OCH_2_Cl) (0.4 mL, 5.3 mmol). The mixture was refluxed for 24 h, cooled to room temperature, filtered, washed with acetone, and the solvent was evaporated. The resulting orange oil was purified using flash column chromatography (petroleum ether/ethyl acetate 9:1) to give (**1a**) as a low melting yellow solid. Yield: 548 mg (80%); ^1^H-NMR (300 MHz, CDCl_3_): δ (ppm) 12.58 (s, 1H, -OH), 7.65 (d, *J* = 8.8 Hz, 1H, H-6), 6.60 (d, *J* = 2.4 Hz, 1H, H-3), 6.56 (dd, *J* = 8.8, 2.4 Hz, 1H, H-5), 5.22 (s, 2H, 2 × -OCH_2_-), 3.50 (s, 3H, 3 × -OCH_3_), 2.59 (s, 3H, 3 × -CH_3_).

Note: Chloromethyl methylether is a highly toxic compound and should not be ingested or inhaled. All contact with this material should be avoided. It is a flammable material, corrosive to eyes and skin on contact. It is a possible carcinogen. It is lachrymator; Heat and sources of ignition should be avoided.

*3,4-bis(methoxymethoxy)-benzaldehyde* (**2a**) [17]: Prepared following the procedure described for the synthesis of acetophenone **1a**, compound **2a** was prepared from 3,4-dihydroxy-benzaldehyde (500 mg, 3.6 mmol). After evaporation of the solvent, the oily resulting product was purified using flash column chromatography (petroleum ether/ethyl acetate 8:2 (2 × 100 mL) and 7:3 (2 × 100 mL) with dry packing to give benzaldehyde **2a** as a low melting white solid. Yield: 503.6 mg (62%). mp 61.2–62 °C; ^1^H-NMR (300 MHz, CDCl_3_): δ (ppm) 9.82 (s, 1H, HC = O), 7.64 (d, *J* = 1.9 Hz, 1H, H-3), 7.47 (dd, *J* = 8.3, 1.9 Hz, 1H, H-2), 7.23 (d, *J* = 1.0 Hz, 1H, H-6), 5.30 (s, 2H, -OCH_2_-), 5.27 (s, 2H, -OCH_2_-), 3.51 (s, 3H, -COCH_3_), 3.51 (s, 3H, -COCH_3_).

#### 3.1.1. General Procedure for the Synthesis of Chalcones **3a**–**3l**

(1) Method A

To a stirred solution of the appropriate 2-hydroxy-acetophenone (1 eq) and a substituted benzaldehyde (1 eq) in ethanol is added KOH (20% *w/v* aqueous solution). The mixture is refluxed for 4 h or stirred in room temperature for 24 h. The completion of the reaction is monitored by TLC. At the end of the reaction, the mixture is cooled to 0 °C (ice-water bath) and acidified with HCL (10% *v/v* aqueous solution). The yellow precipitate formed is filtered and washed with 10% aqueous HCl solution.

(2) Method B

To a stirred solution of the appropriate 2-hydroxy-acetophenone (1 eq) and a substituted benzaldehyde (1 eq) in dry dimethylformamide (DMF) is added NaH (60% *w/w*) and the mixture is stirred at room temperature for 24 h. The completion of the reaction is monitored by TLC. At the end of the reaction, the mixture is cooled to 0 °C (ice-water bath) and acidified with HCl (10% *v/v* aqueous solution). An orange-yellow oil is formed and the mixture is extracted with diethyl ether; the extracts are dried with Na_2_SO_4_ and the solvent is evaporated under reduced pressure to give the chalcone as an orange-yellow solid.

Note: NaH dispersed in oil was used for the reaction. Special attention must be paid when using NaH, as it can ignite in air, especially upon contact with water to release hydrogen, which is also flammable.

*2′-hydroxy-4′,3,4 -methoxymethoxy-chalcone* (**3a**): Prepared according to the general procedure (method B) starting from 2-hydroxy-4-(methoxymethoxy)-acetophenone (**1a**) (418 mg, 2.1 mmol) and 3,4-bis(methoxymethoxy)benzaldehyde (**2a**) (504 mg, 2.1 mmol), dissolved in 4.5 mL dry DMF and NaH (60% *w/w*, 255.43 mg). After the work-up procedure, the product was obtained upon recrystallization from hexane/ethyl acetate as an orange-yellow powder. The orange-yellow solid was recrystallized from hexane/ethyl acetate. Yield: 342 mg (40%). m.p. 98–100 °C (ref. [73] m.p.107.8 °C); ^1^H-NMR (300 MHz, CDCl_3_): δ (ppm) 13.30 (s, 1H, OH), 7.84 (d, *J* = 8.7 Hz, 1H, H-6′), 7.81 (d, *J* = 15.3 Hz, 1H, Hb), 7.43 (m, 2H, H-5, Ha), 7.29 (m, 1H, H-2), 7.19 (d, *J* = 8.1 Hz, 1H, H-6), 6.63 (d, *J* = 2.1 Hz, 1H, H-3′), 6.59 (dd, *J* = 8.7, 1.8 Hz, 1H, H-5′), 5.96 (br, 4H, 2 × -OCH_2_O-), 5.22 (s, 2H, -OCH_2_O-), 3.56 (s, 3H, -CH_3_), 3.53 (s, 3H, -CH_3_), 3.50 (s, 3H, -CH_3_); ^13^C-NMR (150 MHz, CDCl_3_): δ (ppm) 191.95, 166.13, 163.51, 149.62, 147.42, 144.35, 131.28, 129.19, 124.11, 118.73, 116.18, 116.10, 114.95, 108.116, 103.93, 95.54, 95.10, 94.01, 56.37, 56.35, 56.33.

*2′-hydroxy-2,4,5-trimethoxy-chalcone* (**3b**): Prepared according to the general procedure (method A) starting from 2-hydroxy-acetophenone (1b) (500 mg, 3.7 mmol) and 2,4,5-trimethoxy-benzaldehyde (2b) (721 mg, 3.7 mmol), dissolved in 6 mL ethanol and 3.1 mL of 20% *w/v* aqueous solution KOH. The mixture was refluxed for 4 h. After the work-up procedure, the product was obtained upon recrystallization from hexane/ethyl acetate as an orange-yellow powder. Yield: 546.3 mg (47%). m.p. 129–130 °C; (ref. [74] m.p. 124–125 °C); ^1^H-NMR (300 MHz, CDCl3): δ (ppm) 13.04 (s, 1H, -OH), 8.22 (d, *J* = 15.3 Hz, 1H, Hb), 7.93 (dd, *J* = 8.1, 1.5 Hz, 1H, H-6′), 7.62 (d, J = 15.3 Hz, 1H, Ha), 7.48 (ddd, *J* = 8.5, 7.2, 1.6 Hz, 1H, H-4′), 7.13 (s, 1H, H-6), 7.02 (dd, *J* = 8.4, 0.9 Hz, 1H, H-3′), 6.94 (ddd, *J*= 8.5, 7.2, 1.2 Hz, 1H, H-5′), 6.54 (s, 1H, H-3), 3.98 (s, 3H, -OCH_3_), 3.96 (s, 3H, -OCH_3_), 3.94 (s, 3H, -OCH_3_).

*2′-hydroxy-4′-methoxymethoxy-2,4,5-trimethoxy-chalcone* (**3c**): Prepared according to the general procedure (method B) starting from 2-hydroxy-4-(methoxymethoxy)-acetophenone (**1a**) (60 mg, 0.3 mmol) and 2,4,5-trimethoxy-benzaldehyde (**2b**) (60 mg, 0.3 mmol), dissolved in 0.8 mL dry DMF and NaH (60% *w/w*, 36.67 mg). After the work-up procedure, the residue was purified using flash column chromatography (petroleum ether/ethyl acetate 7.5:2.5) to give **3c** as an orange solid. Yield: 145 mg (39%). mp 86.2–86.8 °C; ^1^H-NMR (600 MHz, CDCl_3_): δ (ppm) 13.51 (s, 1H, -OH), 8.17 (d, *J* = 15.6 Hz, 1H, Hb), 7.85 (d, *J* = 8.7 Hz, 1H, H-6′), 7.54 (d, *J* = 15.6 Hz, 1H, Ha), 7.11 (s, 1H, H- 6), 6.63 (d, *J*= 1.8 Hz, 1H, H-3′), 6.58 (dd, *J* = 8.4, 1.8 Hz, 1H, H-5′), 6.52 (s, 1H, H-3), 5.22 (s, 2H, -OCH_2_O-), 3.95 (s, 3H, -OCH_3_), 3.92 (br, 6H, 2 × -OCH_3_), 3.49 (s, 3H, -OCH_2_OCH_3_); ^13^C-NMR (150 MHz, DMSO-d_6_): δ (ppm) 192.63, 166.22, 163.41, 155.08, 152.93, 143.45, 140.21, 131.34, 118.24, 115.53, 115.33, 112.02, 108.07, 104.09, 96.97, 94.17, 56.77, 56.52, 56.50, 56.23; HR-MS *m/z* (neg): 373.1291 C_20_H_22_O_4_ (calcd. 374.1366).

*5′-chloro-3,4-dimethoxy-2′-hydroxy-chalcone* (**3d**): Prepared according to the general procedure (method A) starting from 5-chloro-2-hydroxyacetophenone (**1c**) (500 mg, 2.9 mmol) and 3,4-dimethoxy-benzaldehyde (**2c**) (486.9 mg, 2.9 mmol), dissolved in 7.2 mL ethanol and 2.5 mL of 20% *w/v* aqueous solution KOH. The mixture was stirred at room temperature for 24 h. After the work-up procedure, the product was obtained upon recrystallization from methanol/dichloromethane as a yellow powder. Yield: 630 mg (72%); m.p. 170–172 °C (ref. [73] m.p. 167–168 °C]; ^1^H-NMR (600 MHz, DMSO-d_6_): δ (ppm) 12.56 (s, 1H, -OH), 8.25 (d, *J* = 2.6 Hz, 1H, H-6′), 7.87 (d, *J* = 15.0 Hz, 1H, Hb), 7.81 (d, *J* = 15.6 Hz, 1H, Ha), 7.57 (m, 2H, Ar), 7.47 (dd, *J* = 7.8, 1.2 Hz, 1H, Ar), 7.04 (m, 2H, Ar), 3.86 (s, 3H, -OCH_3_), 3.83 (s, 3H, -OCH_3_); ^13^C-NMR (150 MHz, DMSO-d_6_): δ (ppm) 192.45, 160.08, 151.76, 148.94, 146.17, 135.45, 129.38, 127.09, 124.43, 122.70, 122.14, 119.71, 119.11, 111.61, 111.15, 55.83, 55.60.

*5′-chloro-2,3-dimethoxy-2′-hydroxy-chalcone* (**3e**) [31]: Prepared according to the general procedure (method A) starting from 5-chloro-2-hydroxy-acetophenone (**1c**) (341 mg, 2.0 mmol) and 2,3-dimethoxy-benzaldehyde (**2d**) (330 mg, 2.0 mmol), dissolved in 5 mL ethanol and 1.6 mL of 20% *w/v* aqueous solution of KOH. The mixture was stirred at room temperature for 24 h. After the work-up procedure, the product was obtained upon recrystallization from methanol/dichloromethane as a yellow powder. Yield: 390 mg (50%). mp 133–134 °C; ^1^H-NMR (600 MHz, DMSO-d_6_): δ (ppm) 12.18 (s, 1H, -OH), 8.18 (d, *J*= 2.4 Hz, 1H, H-6′), 8.06 (d, *J* = 15.6 Hz, 1H, Hb), 7.75 (d, *J* = 15.6 Hz, 1H, Ha), 7.66 (dd, *J* = 7.2, 1.8 Hz, 1H, H-6), 7.57 (dd, *J* = 9.0, 2.4 Hz, 1H, H-4′), 7.17 (m, 2H, H-4, H-5), 7.04 (d, *J* = 9.0 Hz, 1H, H-3′), 3.85 (s, 3H, -OCH_3_), 3.80 (s, 3H, -OCH_3_); ^13^C-NMR (75 MHz, DMSO-d_6_): δ (ppm) 192.30, 162.50, 155.80, 148.30, 138.60, 135.10, 129.70, 127.80, 125.50, 124.10, 123.60, 122.90, 115.60, 112.40, 112.16, 60.80, 55.58.

*5′-bromo-3,4-dimethoxy-2′-hydroxy-chalcone* (**3f**) [41]: Prepared according to the general procedure (method A) starting from 5-bromo-2-hydroxy-acetophenone (**1d**) (500 mg, 2.0 mmol) and 3,4-dimethoxy-benzaldehyde (**2c**) (330 mg, 2.0 mmol), dissolved in 5 mL ethanol and 1.6 mL of 20% *w/v* aqueous solution of KOH. The mixture was stirred at room temperature for 24 h. After the work-up procedure, the product was obtained upon recrystallization from methanol/dichloromethane as a yellow powder. Yield: 300 mg (50%). mp 178–179 °C; ^1^H-NMR (600 MHz, DMSO-d_6_): δ (ppm) 12.56 (s, 1H, -OH), 8.33 (s, 1H, H-6′), 7.86 (d, *J* = 15.6 Hz, 1H, Hb), 7.80 (d, *J* = 15.0 Hz, 1H, Ha), 7.68 (dd, *J* = 9.0, 2.4 Hz, 1H, H-4′), 7.55 (d, *J*= 1.8 Hz, 1H, H-2), 7.47 (dd, *J* = 8.4, 1.8 Hz, 1H, H-6), 7.04 (d, *J* = 8.4 Hz, 1H, H-5), 6.97 (d, *J* = 8.4 Hz, 1H, H-3′), 3.86 (s, 3H, -OCH_3_), 3.83 (s, 3H, -OCH_3_); ^13^C-NMR (75 MHz, DMSO-d_6_): δ (ppm) 192.90, 160.50, 152.10, 149.60, 146.30, 138.40, 132.30, 127.10, 124.50, 123.00, 119.30, 112.02, 111.58, 111.30, 110.20, 55.80, 55.50.

*3′,5′-dibromo-3,4-dimetoxy-2′-hydroxy-chalcone* (**3g**) [41]: Prepared according to the general procedure (method A) starting from 3,5-dibromo-2-hydroxy-acetophenone (**1e**) (500 mg, 1.7 mmol) and 3,4-dimethoxy-benzaldehyde (**2c**) (282.5 mg, 1.7 mmol), dissolved in 5 mL ethanol and 1.4 mL of 20% *w/v* aqueous solution of KOH. The mixture was stirred at room temperature for 24 h. After the work-up procedure, the product was obtained upon recrystallization from methanol/dichloromethane as a yellow powder. Yield: 470 mg (63%). mp 165–166 °C; ^1^H-NMR (600 MHz, DMSO-d_6_): δ (ppm) 13.75 (s, 1H, -OH), 8.55 (d, *J*= 2.4 Hz, 1H, H-6′), 8.11 (d, *J*= 2.4 Hz, 1H, H-4′), 7.95 (d, *J* = 15.6 Hz, 1H, Hb), 7.91 (d, *J* = 15.6 Hz, 1H, Ha), 7.60 (d, *J*= 1.8 Hz, 1H, H-2), 7.53 (dd, *J* = 8.4, 1.8 Hz, Hz, 1H, H-6), 7.06 (d, *J* = 8.4 Hz, 1H, H-5), 3.87 (s, 3H, -OCH_3_), 3.84 (s, 3H, -OCH_3_); ^13^C-NMR (75 MHz, DMSO-d_6_): δ (ppm) 195.30, 161.60, 155.20, 152.00, 150.80, 143.50, 135.10, 130.20, 128.20, 121.00, 119.20, 115.50, 114.70, 114.50, 113.00, 58.80, 58.60.

*5′-bromo-3,4-dimethoxymethoxy-2′-hydroxy-chalcone* (**3h**): Prepared according to the general procedure (method A) starting from 5-bromo-2-hydroxy-acetophenone (**1d**) (430 mg, 2.0 mmol) and 3,4-dimethoxymethoxy-benzaldehyde (**2a**) (500 mg, 2.0 mmol), dissolved in 5 mL ethanol and 1.6 mL of 20% *w/v* aqueous solution of KOH. The mixture was stirred at room temperature for 24 h. After the work-up procedure, the product was purified using flash column chromatography (petroleum ether/ethyl acetate 8:2) to give **3h** as a yellow solid. Yield: 38 mg (22%). mp 105–106 °C; ^1^H-NMR (600 MHz, DMSO-d_6_): δ (ppm) 12.37 (s, 1H, -OH), 8.28 (d, *J* = 2.4 Hz, 1H, H-6′), 7.83 (d, *J*= 15.0 Hz, 1H, Hb), 7.75 (d, *J*= 15.6 Hz, 1H, Ha), 7.68 (dd, *J* = 9.0, 2.4 Hz, 1H, H-4′), 7.66 (d, *J* = 1.8 Hz, 1H, H-2), 7.55 (dd, *J* = 8.4, 1.2 Hz, 1H, H-6), 7.18 (d, *J* = 8.4 Hz, 1H, H-3′), 6.98 (d, *J* = 8.4 z, 1H, H-5), 5.29 (s, 4H, 2 × -OCH_2_-), 3.44 (s, 3H, -OCH_3_), 3.41 (s, 3H, -OCH_3_); ^13^C-NMR (150z MHz, DMSO-d_6_): δ (ppm) 192.25, 160.19, 149.87, 146.65, 145.39, 138.05, 132.34, 128.46, 124.96, 123.24, 120.48, 120.06, 117.53, 116.28, 110.28, 94.92, 94.42, 55.94, 55.91; HR-MS *m/z* (neg): 421.0288 C_19_H_19_BrO_6_ (calcd. 422.0365).

*3′,5′-bromo-3,4-dimethoxymethoxy-2′-hydroxy-chalcone* (**3i**): Prepared according to the general procedure (method A) starting from 3,5-dibromo-2-hydroxy-acetophenone (**1e**) (588 mg, 2.0 mmol) and 3,4-dimethoxymethoxy-benzaldehyde (**2a**) (500 mg, 2.0 mmol), dissolved in 5 mL ethanol and KOH 1.6 mL of 20% *w/v* aqueous solution of KOH. The mixture was stirred at room temperature for 24 h. After the work-up procedure the product was obtained as a yellow powder. Yield: 38 g (22%). mp 134–135 °C (ref. [46] m.p. 129–131 °C); ^1^H-NMR (600 MHz, CDCl_3_): δ (ppm) 13.63 (s, 1H, -OH), 7.98 (d, *J* = 2.4 Hz, 1H, H-6′), 7.92 (d, *J* = 15.0 Hz, 1H, Hb), 7.87 (d, *J* = 2.4 Hz, 1H, H-4′), 7.48 (d, *J* = 1.8 Hz, 1H, H-2), 7.39 (d, *J* = 15.0 Hz, 1H, Ha), 7.33 (dd, *J* = 8.4, 1.2 Hz, 1H, H-6), 7.22 (d, *J* = 8.4 Hz, 1H, H-5), 5.31 (br, 4H, 2 × -O-CH_2_-O-), 3.56 (s, 3H, -OCH_3_), 3.53 (s, 3H, -OCH_3_).

*4-carboxy-2′-hydroxy-chalcone* (**3j**) [41]: Prepared according to the general procedure (method A) starting from 2-hydroxy-acetophenone (**1b**) (1 g, 7.3 mmol) and 4-carboxy-benzaldehyde (**2g**) (1.102 g, 7.3 mmol), dissolved in 18 mL ethanol and 6 mL of 20% *w/v* aqueous solution of KOH. The mixture was stirred at room temperature for 24 h. After the work-up procedure, the product was obtained upon recrystallization from methanol/dichloromethane as a yellow powder. Yield: 813 mg (63%). mp 253–255 °C; ^1^H-NMR (600 MHz, DMSO-d_6_): δ (ppm) 13.13 (s, 1H, -COOH), 12.31 (s, 1H, -OH), 8.22 (d, *J* = 8.2 Hz, 1H, H-6′), 8.10 (d, *J* = 15.6 Hz, 1H, Hb), 7.99 (m, 4H, H-2, H-3, H-5, H-6), 7.84 (d, *J* = 15.6 Hz, 1H, Ha), 7.56 (t, *J* = 7.8 Hz, 1H, H-5′), 6.99 (m, 2H, H-3′, H-4′); ^13^C-NMR (150 MHz, DMSO-d_6_): δ (ppm) 192.51, 166.33, 161.30, 142.98, 138.51, 136.47, 132.41, 131.08, 129.86, 129.26, 124.44, 121.19, 119.5, 118.05.

*4-carboxy-5′-chloro-2′-hydroxy-chalcone* (**3k**) [48]: Prepared according to the general procedure (method A) starting from 5-chloro-2-hydroxy-acetophenone (**1c**) (500 mg, 2.9 mmol) and 4-carboxy-benzaldehyde (**2g**) (440 mg, 2.9 mmol), dissolved in 7.2 mL ethanol and 2.5 mL of 20% *w/v* aqueous solution of KOH. The mixture was stirred at room temperature for 24 h. After the work-up procedure, the product was obtained upon recrystallization from methanol/dichloromethane as a yellow powder. Yield: 710 mg (85%). mp 284–286 °C; ^1^H-NMR (600 MHz, DMSO-d_6_): δ (ppm) 13.15 (s, 1H, -COOH), 12.15 (s, 1H, -OH), 8.23 (d, *J* = 3.0 Hz, 1H, H-6′), 8.08 (d, *J* = 15.6 Hz, 1H, Hb), 8.01 (m, 4H, H-2, H-3, H-5, H-6), 7.84 (d, *J* = 15.6 Hz, Ha), 7.58 (dd, *J* = 8.4, 2.4 Hz, 1H, H-4′), 7.04 (d, *J* = 9.0 Hz, 1H, H-3′); ^13^C-NMR (150 MHz, DMSO-d_6_): δ (ppm) 192.64, 167.22, 160.16, 143.91, 138.88, 136.05, 132.83, 130.18, 130.07, 129.67, 124.91, 123.43, 123.11, 120.22.

*2′-hydroxy-chalcone* (**3l**): Prepared according to the general procedure (method A) starting from 2-hydroxy-acetophenone (**1b**) (500 mg, 3.7 mmol) and benzaldehyde (**2h**) (392.6 mg, 3.7 mmol), dissolved in 6 mL ethanol and 3.1 mL of 20% *w/v* aqueous solution KOH. The mixture was stirred at room temperature for 24 h. After the work-up procedure, the product was obtained upon recrystallization from methanol/dichloromethane as a yellow powder. Yield: 697 mg (84%); m.p. 85–86 °C (ref. [49] m.p. 82–85 °C); ^1^H-NMR (600 MHz, CDCl_3_): δ (ppm) 12.83 (s, 1H, -OH), 7.93 (m, 2H, aromatic H), 7.67 (m, 4H, aromatic H and Hb), 7.51 (ddd, *J* = 8.4, 7.2, 1.2 Hz, 1H, aromatic H), 7.45 (m, 3H, aromatic H and Ha), 7.04 (d, *J* = 8.4 Hz, 1H, aromatic H), 6.96 (t, *J* = 7.2 Hz, 1H aromatic H).

#### 3.1.2. General Procedure for the Synthesis of Chalcones **4a** and **4b**

Chalcones **4a** and **4b** were produced by removing the MOM protecting group. The corresponding methoxymethoxy-chalcone wasdissolved in methanol and 10% aqueous HCl was added dropwise. The mixture was stirred at 65 °C for 15 min. The reaction was monitored by TLC. After completion of the reaction, the mixture was diluted with water and extracted with ethyl acetate. The organic extracts were dried with Na_2_SO_4_ and the solvent was evaporated under reduced pressure to give the chalcone as a solid product.

*2′,3,4,4′-tetrahydroxy-chalcone (Butein)* (**4a**): Prepared according to the general procedure, chalcone (**3a**) (47.5 mg, 0.1 mmol) was dissolved in 2.2 mL methanol followed by dropwise addition HCl (10% aqueous solution, 1 mL). The mixture was refluxed for 15 min, until a clear red solution was obtained. After the work-up procedure, a red solid was acquired. Yield: 22.4 mg (70%). m.p. > 180 °C; ^1^H-NMR (300 MHz, DMSO-d_6_): δ (ppm) 13.53 (s, 1H, OH-2′), 10.60 (s, 1H, OH-4′), 9.71 (s, 1H, OH-4), 9.06 (s, 1H, OH-3), 8.11 (d, *J* = 8.9 Hz, 1H, H-6′), 7.63 (m, 2H, Ha, Hb), 7.25 (d, *J* = 1.8 Hz, 1H, H-3′), 7.18 (dd, *J* = 8.1, 1.8 Hz, 1H, H-5′), 6.79 (d, *J* = 8.1 Hz, 1H, H-5), 6.38 (dd, *J* = 8.7, 2.1 Hz, 1H, H-6), 6.25 (d, *J* = 2.4 Hz, 1H, H-2); MS (ESI) (positive): *m/z* = 273.16 (50%) [M + 1]^+^, 206.61 (77%) [M − 4 × -OH + 2]^+^; MS (ESI) (negative): *m/z* = 271.2 (100%) [M − 1]^+^, 542.79 (92%) [2 × (M − 1)]^+^.

*5′-bromo-2′,3,4-trihydroxy-chalcone* (**4b**): Prepared according to the general procedure, chalcone (**3h**) (200 mg, 0.5 mmol) was dissolved in 9.8 mL methanol followed by dropwise addition of HCL (10% aqueous solution, 4 mL). The mixture was refluxed for 15 min. After the work-up procedure, the product was obtained upon recrystallization from methanol/dichloromethane. Yield: 500 mg (50%). mp 198–199 °C; ^1^H-NMR (600 MHz, DMSO-d_6_): δ (ppm) 12.59 (s, 1H, -OH), 9.88 (brs, 1H, Ar-OH), 9.14 (brs, 1H, Ar-OH), 8.34 (d, *J*= 2.4 Hz, 1H, H-6′), 7.71 (m, 2H, Ha, Hb), 7.66 (dd, *J* = 8.4, 2.4 Hz, 1H, H-4′), 7.34 (d, *J*= 1.2 Hz, 1H, H-2), 7.24 (dd, *J* = 8.4, 1.8 Hz, 1H, H-6), 6.96 (d, *J* = 9.0 Hz, 1H, H-3′), 6.83 (d, *J* = 7.8 Hz, 1H, H-5); ^13^C-NMR (150 MHz, DMSO-d_6_): δ (ppm) 192.16, 160.46, 149.47, 146.70, 145.68, 137.98, 132.36, 126.07, 123.18, 122.98, 120.04, 118.04, 116.01, 115.75, 110.25; HR-MS *m/z* (neg): 332.9763 C_15_H_11_BrO_4_ (calcd. 333.9841).

#### 3.1.3. Synthetic Procedure of 4-Methoxycarbonyl-2′-Hydroxy-Chalcone (**5**)

Chalcone (**3j**) (500 mg, 1.9 mmol) was added to methanol (50 mL), followed by dropwise addition of H_2_SO_4_ (5 mL). The mixture was stirred at 70 °C for 24 h. The reaction was monitored by TLC. After completion of the reaction, the solvent was evaporated under reduced pressure and the residue was cooled at −19 °C. The precipitate formed was filtered and washed with methanol. Yield: 317 mg (60%). mp: 128–131 °C; ^1^H-NMR (600 MHz, DMSO-d_6_): δ (ppm) 12.31 (s, 1H, -OH), 8.24 (dd, *J* = 8.4, 1.2 Hz, 1H, H-6′), 8.14 (d, *J* = 15.6 Hz, 1H, Hb), 8.04 (m, 4H, H-2,H-3,H-5,H-6), 7.86 (d, *J* = 15.6 Hz, 1H, Ha), 7.58 (ddd, *J* = 8.4, 7.8, 1.8 Hz, 1H, H-4′), 7.02 (m, 2H, H-3′, H-5′), 3.88 (s, 3H, -COOCH_3_); ^13^C-NMR (150 MHz, DMSO-d_6_): δ (ppm) 193.3, 165.7, 161.7, 142.8, 139.0, 136.5, 131.0, 129.6, 129.2, 124.6, 121.0, 119.2, 117.7, 109.6, 52.3; HR-MS *m/z* (neg): 281.0809 C_17_H_14_O_4_ (calcd. 282.0892).

#### 3.1.4. General Procedure for the Synthesis of Aurones **6a** and **6b**

To a solution of mercuric acetate (1.25 eq) in pyridine was added the desirable chalcone (1 eq) at room temperature and the mixture was stirred at 110 °C for 1 h. The reaction was monitored by TLC. The cooled reaction mixture was poured into ice cold water and acidified with HCl (10% aqueous solution). The precipitated solid was extracted with dichloromethane, the extracts were dried (Na_2_SO_4_) and the solvent was evaporated to give a solid which was further purified by recrystallization from methanol/dichloromethane.

Note: Pyridine is a harmful reactant and should be handled with care as it can be harmful in case of inhalation, ingestion, or when in contact with the skin. Pyridine is also known to reduce male fertility. Pyridine has a very noticeable and unpleasant smell, therefore, good ventilation is essential, when used. To neutralize pyridine addition of a weak acid, such as 10% aqueous HCl, at 0 °C is suggested.

Mercuric acetate is a harmful reagent. It is fatal if swallowed, in contact with skin or if inhaled. It may cause damage to organs through prolonged or repeated exposure. It is very toxic to aquatic life with long lasting effects. Protective gloves/protective clothing should be worn throughout the period of handling this reagent.

*3′,4′,6-tri(methoxymethoxy)-aurone* (**6a**): Prepared according to the general procedure starting from chalcone (**3a**) (140.4 mg, 0.3 mmol), dissolved in 3.5 mL pyridine. After the work-up procedure, the product was obtained upon recrystallization from methanol/dichloromethane as a yellow solid. Yield: 84 mg (60%). mp 140.2–141.4 °C; ^1^H-NMR (300 MHz, CDCl_3_): δ (ppm) 7.71 (m, 2H, H-4, H-10), 7.55 (d, *J* = 8.4 Hz, 1H, H-5), 7.22 (d, *J*= 8.7 Hz, 1H, H- 5′), 6.95 (s, 1H, H-7), 6.86 (d, *J* = 8.4 Hz, 1H, H-6′), 6.78 (s, 1H, H-2′), 5.31 (s, 6H, 3 × -OCH_2_O-), 3.57 (s, 3H, -CH_2_OCH_3_), 3.53 (br, 6H, 2 × -CH_2_OCH_3_); ^13^C-NMR (150 MHz, CDCl_3_): δ (ppm) 183.16, 168.09, 164.82, 149.03, 147.29, 147.22, 126.98, 126.84, 125.93, 119.99, 116.47, 116.04, 113.27, 112.24, 99.48, 95.90, 95.29, 94.59, 56.58, 56.54, 56.48; HR-MS *m/z* (pos): 403.1352 C_21_H_22_O_8_ (calcd. 402.1315).

*2′,4′,5′-trimethoxy-aurone* (**6b**): Prepared according to the general procedure starting from chalcone (**3b**) (150 mg, 0.4 mmol) and mercury acetate (184.25 mg, 0.6 mmol) dissolved in 4.8 mL pyridine. After the work-up procedure, the product was obtained upon recrystallization from methanol/dichloromethane as a yellow solid. Yield: 112.7 mg (74%). mp 181.2–183.4 °C (ref. [50] m.p. 191–193 °C); ^1^H-NMR (300 MHz, DMSO-d_6_): δ (ppm) 7.78 (s, 1H, H-10), 7.75 (m, 2H, H-4, H-5) 7.56 (d, *J* = 8.4 Hz, 1H, H-7), 7.28 (t, *J* = 7.5 Hz, 1H, H-6), 7.17 (s, 1H, H- 6′), 6.77 (s, 1H, H-3′), 3.91 (br, 6H, 2 × -OCH_3_), 3.82 (s, 3H, -OCH_3_).

#### 3.1.5. Synthetic Procedure of 3′,4′,6-Trihydroxy-Aurone (Sulfuretin) (**7a**)

Aurone (**7a**) was prepared via deprotection reaction of aurone (**6a**). Aurone (**6a**) (48.2 mg, 0.1 mmol) was added to 2.25 mL methanol followed by dropwise addition of HCl (10% aqueous solution, 1 mL). The mixture was refluxed for 15 min, until a clear orange solution was obtained. The reaction was monitored by TLC. After completion of the reaction, the mixture was diluted with water and extracted with ethyl acetate. The organic extracts were dried with Na_2_SO_4_ and the solvent was evaporated under reduced pressure to give the product as an orange solid. Yield: 29.6 mg (92%). mp > 250 °C; ^1^H-NMR (300 MHz, DMSO-d_6_): δ (ppm) 11.04 (s, 1H, OH-3′), 9.60 (s, 1H, OH-4′), 9.23 (s, 1H, OH-6), 7.57 (d, *J* = 8.1 Hz, 1H, H-4), 7.42 (d, *J* = 1.8 Hz, 1H, H-7), 7.22 (dd, *J* = 8.4, 1.8 Hz, 1H, H-5), 6.81 (d, *J* = 8.4 Hz, 1H, H-5′), 6.72 (d, *J* = 1.5 Hz, 1H, H-2′), 6.68 (dd, *J* = 8.4, 1.8 Hz, 1H, H-6′), 6.61 (s, 1H, H-10); ^13^C-NMR (150 MHz, CD_3_OD-d_4_): δ (ppm) 184.47, 169.77, 168.24, 149.37, 147.69, 146.70, 126.83, 126.40, 125.53, 118.94, 116.66, 114.90, 114.70, 114.03; MS (ESI) (positive): *m/z* = 271.14 (67%) [M + 1]^+^, 562.75 (42%) [2 × M + Na]^+^; MS (ESI) (negative): *m/z* = 269.23 (33%) [M − 1]^+^, 538.86 (100%) [2 × (M − 1)]^+^.

#### 3.1.6. Synthetic Procedure of 2′,4′,5′-Trihydroxy-Aurone (**7b**)

Aurone (**7b**) was prepared via deprotection reaction of aurone (**6b**). Aurone (**6b**) (50 mg, 0.2 mmol) was added to 2 mL dry dichloromethane followed by addition of BBr_3_ (2.4 mL, 2.4 mmol). The mixture was stirred for 2 h at 0 °C, until a clear orange solution was obtained. The reaction was monitored by TLC. After completion of the reaction, the resulting mixture was poured into ice-cold water and left to stand for 25 min. The residue was extracted with ethyl acetate, the organic extracts were dried with Na_2_SO_4_, and the solvent was evaporated under reduced pressure to give the product as a red solid. Yield: 26.8 mg (62%). mp > 250 °C; ^1^H-NMR (300 MHz, CD_3_OD-d_4_): δ (ppm) 7.74 (s, 1H, H-10), 7.70 (m, 2H, H-4, H-5), 7.44 (s, 1H, H-6′), 7.37 (d, *J* = 8.4 Hz, 1H, H-7), 7.247. (t, *J* = 7.2 Hz, 1H, H-6), 6.38 (s, 1H, H-3′); MS (ESI): *m/z* = 271.2 (55%) [M + 1]^+^, 255.3 (100%) [M − 1 × OH + 1]^+^.

### 3.2. Biological In Vitro Assays

Each in vitro experiment was performed at least in triplicate and the standard deviation of absorbance was less than 10% of the mean. For the in vitro assays, a stock solution (1% DMSO in the appropriate buffer with the tested compound diluted under sonification) was prepared from which several dilutions were made with the appropriate buffer.

#### 3.2.1. Determination of the Reducing Activity of the Stable Radical 2,2-Diphenyl-1-Picrylhydrazyl (DPPH)

To an ethanolic solution of DPPH (100 μM) in absolute ethanol, an equal volume of the compounds dissolved in DMSO was added (100 μM). The mixture was shaken vigorously and allowed to stand for 20 or 60 min; absorbance at 517 nm was determined spectrophotometrically, and the percentage of activity was calculated. All tests were undertaken on three replicates, and the results presented in Table 2 were averaged and compared with the appropriate standard nordihydroguaiaretic acid (NDGA) [61].

#### 3.2.2. Inhibition of Linoleic Acid Lipid Peroxidation

Production of conjugated diene hydroperoxide by oxidation of sodium linoleate in an aqueous solution is monitored at 234 nm. 2,2′-Azobis(2-amidinopropane) dihydrochloride (AAPH) was used as a free radical initiator. Ten microliters of the 16 mM linoleic acid sodium salt solution was added to the UV cuvette containing 0.93 mL of 0.05 M phosphate buffer, pH 7.4 prethermostated at 37 °C. The oxidation reaction was initiated at 37 °C under air by the addition of 50 μL of 40 mM AAPH solution. Oxidation was carried out in the presence of the synthesized compounds (10 μL, from a stock solution of 10 mM in DMSO). In the assay without antioxidant, lipid oxidation was measured in the presence of the same level of DMSO. The rate of oxidation at 37 °C was monitored by recording the increase in absorption at 234 nm caused by conjugated diene hydroperoxides and compared with the appropriate standard trolox [61].

#### 3.2.3. Competition of the Tested Compounds with DMSO for Hydroxyl Radicals

The hydroxyl radicals generated by the Fe^3+^ / ascorbic acid system, were detected by the determination of formaldehyde produced from the oxidation of DMSO (Table 2). A negative control using the same % DMSO was used. Trolox was used as reference compound [75].

#### 3.2.4. ABTS^+^—Decolorization Assay for Antioxidant Activity

In order to produce the ABTS radical cation (ABTS^+·^), ABTS stock solution in water (7 mM) was mixed with potassium persulfate (2.45 mM) and left in the dark at room temperature for 12–16 h before use. Our published experimental technique was used (1). The results are recorded after 1 min of the mixing solutions at 734 nm. The results were compared to the appropriate standard inhibitor Trolox [75].

#### 3.2.5. Soybean LOX Inhibition Study In Vitro

The tested compounds dissolved in DMSO were incubated at room temperature with sodium linoleate (0.1 mL) and 0.2 mL of enzyme solution (1/9 × 10^−f^ *w/v* in saline) in Tris buffer pH 9. The conversion of sodium linoleate to 13-hydroperoxylinoleic acid at 234 nm was recorded and compared with the appropriate standard inhibitor NDGA [61].

### 3.3. In Silico Studies

#### 3.3.1. Molecular Docking Studies on Soybean Lipoxygenase Seed Isoform 1 (LOX-1)

The synthesized compounds **3b** and **3c** were primarily sketched in 2D format and prepared at the optimum pH = 7.0 ± 0.5, using LigPrep program of Schrödinger platform. [76] Subsequently, molecular docking simulations were carried out applying the Schrödinger induced fit docking (IFD) protocol [76,77]. The referred protocol, based on glide and prime modules, offers an accurate prediction of ligand favorable binding modes with concomitant structural changes in the receptor (simultaneous flexibility of the side chains, backbone, or both). The crystal structure of soybean LOX-1 (PDB ID: 3PZW) [63] complexed with ferrous iron (Fe^2+^—inactive form) was subjected to protein preparation [78,79]. Particularly, all water molecules were removed, missing residues and hydrogens were added, and restrained energy minimization was followed using OPLS3 force field. Identification of potential allosteric binding sites apart from the iron-binding site and the substrate-binding cavity was performed using SiteMap calculations [68,69,70]. The maximum number of docking poses was set equal to 10, they were visually inspected and their binding modes were analyzed.

#### 3.3.2. Molecular Dynamics Simulations

Molecular dynamics simulations (MD) of the tested ligand-enzyme complexes were performed using the Desmond software of the Schrödinger platform, release 2020-3 [80]. For the examined systems setup, TIP4P was defined as the solvent model and the examined complexes were inserted in an orthorhombic box with volume 850,054 Å^3^. Additionally, OPLS2003 force field was utilized and the systems were neutralized by adding 7 Na^+^ ions. Subsequently, modeled systems were relaxed and then subjected to 10 ns MD simulations in the NPT ensemble class. During the simulation, the temperature was maintained at 300 K using the Nose–Hoover chain thermostat method with a 1.0 ps relaxation time and the pressure was maintained at 1 bar using Martyna–Tobias–Klein barostat method. The root mean square deviation (RMSDs) was calculated for both the docked compounds and Ca atoms of the enzyme, and also interactions and contacts (H-bonds, hydrophobic, ionic, water bridges) of the examined compounds with the LOX-1 residues were monitored throughout the simulation.

## 4. Conclusions

In conclusion, this project reports the synthesis of sixteen 2′-hydroxy chalcone derivatives and four aurone analogues, from which, to our knowledge, compounds **3c**, **3h**, **4b**, **5**, and **6a** are presented in the literature for the first time. The synthesized chalcones and aurones were evaluated for their antioxidant activity using different in vitro techniques, while they were also tested as inhibitors of soybean LOX, as an indication of their anti-inflammatory effect. The bioassays’ results revealed that compound **4b** is the most promising antioxidant agent (82.4% for DPPH scavenging ability and 82.3% in lipid peroxidation inhibition and 100% scavenging of hydroxyl radicals), while analogue **3c** is the most potent LOX inhibitor (IC_50_ = 45 μM). Chalcones **3c** and **3b**, which share the common structural feature of a 2,4,5-trimethoxy substitution at ring B, were further studied to explore their mechanism of action on the soybean LOX-1 enzyme using molecular docking studies and molecular dynamics simulations. The results indicate that these compounds share common binding characteristics and interact with LOX-1 enzyme through allosteric interactions.

## Figures and Tables

**Table 1:** Substituents of all the synthesized chalcone and aurone derivatives.

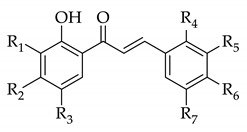 **Generic Chalcone Structure**							
**Compound**	**R_1_**	**R_2_**	**R_3_**	**R_4_**	**R_5_**	**R_6_**	**R_7_**
**3a**	H	OMOM	H	H	OMOM	OMOM	H
**3b**	H	H	H	OCH_3_	H	OCH_3_	OCH_3_
**3c**	H	OMOM	H	OCH_3_	H	OCH_3_	OCH_3_
**3d**	H	H	Cl	H	OCH_3_	OCH_3_	H
**3e**	H	H	Cl	OCH_3_	OCH_3_	H	H
**3f**	H	H	Br	H	OCH_3_	OCH_3_	H
**3g**	Br	H	Br	H	OCH_3_	OCH_3_	H
**3h**	H	H	Br	H	OMOM	OMOM	H
**3i**	Br	H	Br	H	OMOM	OMOM	H
**3j**	H	H	H	H	H	COOH	H
**3k**	H	H	Cl	H	H	COOH	H
**3l**	H	H	H	H	H	H	H
**4a**	H	OH	H	H	OH	OH	H
**4b**	H	H	Br	H	OH	OH	H
**5**	H	H	H	H	H	COOCH_3_	H
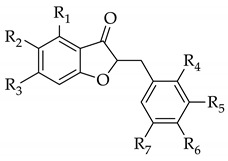 **Generic Aurone Structure**							
**Compound**	**R_1_**	**R_2_**	**R_3_**	**R_4_**	**R_5_**	**R_6_**	**R_7_**
**6a**	H	H	OMOM	H	OMOM	OMOM	H
**6b**	H	H	H	OCH_3_	H	OCH_3_	OCH_3_
**7a**	H	H	OH	H	OH	OH	H
**7b**	H	H	H	OH	H	OH	OH

**Table 2:** In vitro antioxidant evaluation of the synthesized compounds, through the DPPH scavenging ability and the inhibition of linoleic acid assays, as well as antioxidant activity of selected analogues via their ability to scavenge hydroxyl radicals and the ABTS radical cation decolorization assay.

Compound	Interaction with the Free Radical DPPH (%)		Inhibition of Lipid Peroxidation of Linoleic Acid Induced by AAPH Radical (%) 100 μM	HO^·^ (%) 100 µM	ABTS^+·^% 100 µM
	100 μM 20 min	100 μM 60 min			
**3a**	18.9	21.6	11.4	40	93
**3b**	no	no	67.3	no	4
**3c**	no	2.3	39.4	*	*
**3d**	no	no	no	*	*
**3e**	no	no	20.0	*	*
**3f**	no	no	no	*	*
**3g**	no	no	31.0	*	*
**3h**	5.3	6.0	65.6	*	*
**3i**	no	no	93.0	*	*
**3j**	no	no	no	*	*
**3k**	no	no	no	*	*
**3l**	no	no	87.6	*	*
**4a**	95.7	95.3	24.4	68	86
**4b**	82.4	82.0	82.3	100	no
**5**	no	no	100	*	*
**6a**	no	no	18.5	43	86
**6b**	no	no	29.4	no	64
**7a**	89.0	91.2	53.2	no	71
**7b**	100	100	13.4	77	no
**NDGA**	87.0	93.0	*	*	*
**Trolox**	93.0	93.6	92.0	82	93

no: no action under the experimental conditions; *: not tested.

**Table 3:** In vitro % values for DPPH scavenging activity of the most potent compounds in relation to time.

Compound	Interaction with the Free Radical DPPH (%)			
	0 min	3 min	6 min	9 min
**4a**	95.4	95.4	94.9	95.4
**4b**	100	100	100	100
**7a**	92.3	94.4	94.9	94.9
**7b**	98.2	99.3	99.5	99.8

**Table 4:** In vitro % values for AAPH anti-lipid peroxidation activity of the most potent compounds in relation to time.

Compound	Inhibition of Lipid Peroxidation of Linoleic Acid Induced by AAPH Radical (%)			
	0 min	3 min	6 min	9 min
**3i**	95.6	98.6	96.9	99.2
**3l**	87.6	93.2	95.7	94.3
**4b**	81.8	82.6	82.5	81.5
**5**	95.4	96.9	92.5	98.9

**Table 5:** In vitro determination of soybean inhibition; theoretically calculated lipophilicity as ClogP values.

Compound	Inhibition of Soybean Lipoxygenase 100 μM (%) or IC_50_ μM	ClogP ^$^
**3a**	(40.2% at 100 μΜ) ^#^	2.30
**3b**	IC_50_ = 75 μM	3.48
**3c**	IC_50_ = 45 μM	3.08
**3d**	(32.0% at 100 μΜ) ^#^	4.49
**3e**	IC_50_ = 100 μM	4.69
**3f**	(28.0% at 100 μΜ) ^#^	4.64
**3g**	IC_50_ = 68.5 μM	5.33
**3h**	IC_50_ = 55.0 μM	3.86
**3i**	IC_50_ = 67.5 μM	4.54
**3j**	IC_50_ = 100 μM	3.77
**3k**	IC_50_ = 67.5 μM	4.00
**3l**	IC_50_ = 100 μM	3.42
**4a**	(39.3% at 100 μΜ) ^#^	1.65
**4b**	IC_50_ = 70.0 μM	3.14
**5**	IC_50_ = 100 μM	3.39
**6a**	(24.4% at 100 μΜ) ^#^	1.72
**6b**	(13.0% at 100 μΜ) ^#^	2.96
**7a**	(24.5% at 100 μΜ) ^#^	1.97
**7b**	no	1.31
**NDGA**	IC_50_ = 0.45 μΜ	3.92

No: no activity under the reported experimental conditions; ^#^: These compounds were less potent therefore no IC_50_ values were determined for them; ^$^: theoretically calculated ClogP values using the C-QSAR Program, Biobyte [36].

## Data Availability

Not applicable.

## Supplementary Materials

The following are available online: Figure S1: Predicted binding sites of Soybean Lipoxygenase seed isoform 1 (LOX-1), Figure S2: RMSD calculations for (A) all C_a_ enzyme atoms (blue) in LOX-1-Compound **3b** complex and for all atoms of compound **3b** (red), (B) all C_a_ enzyme atoms (blue) in LOX-1-Compound **3c** complex and for all atoms of compound **3c** (red), Figure S3: Categorization of (A) LOX-1-Compound **3b** and (B) LOX-1-Compound 3c interactions, during the molecular dynamics simulation (10 ns). The ^1^H-NMR spectra of all the synthesized compounds and ^13^C-NMR spectra of the novel synthesized compounds.

## Sample Availability

Samples of the compounds are available from the authors.
