# Supplementary material for: Exploring the 2′-Hydroxy-Chalcone Framework for the Development of Dual Antioxidant and Soybean Lipoxygenase Inhibitory Agents
Source: Molecules. 2021 May 8;26(9):2777. doi: 10.3390/molecules26092777 (PMC8125951; doi:10.3390/molecules26092777)

Exploring the 2′-hydroxy-chalcone framework for the development of dual antioxidant and soybean lipoxygenase inhibitory agents

Ioanna Kostopoulou[1], Andromachi Tzani[1], Nestor Ioannis Polyzos[1], Maria-Anna Karadendrou[1], Eftichia Kritsi[2,4], Eleni Pontiki[3], Thalia Liargkova[3], Dimitra Hadjipavlou-Litina[3], Panagiotis Zoumpoulakis[2,4], Anastasia Detsi[1*]

[1] Department of Chemical Sciences, Laboratory of Organic Chemistry, School of Chemical Engineering, National Technical University of Athens, Heroon Polytechniou 9, Zografou Campus, 15780 Athens, Greece; ioanna.th.kostopoulou@gmail.com (I.K.); andromachi.tzani@gmail.com (A.T.); nestor_polyzos@hotmail.com (N.I.P.); markarad96@gmail.com (M.A.K.)

[2] Institute of Chemical Biology, National Hellenic Research Foundation, 48, Vas. Constantinou Avenue, 11635 Athens, Greece; ekritsi@eie.gr (E.K.)

[3] Laboratory of Pharmaceutical Chemistry, School of Pharmacy, Faculty of Health Sciences, Aristotle University of Thessaloniki, Thessaloniki 54124, Greece; epontiki@pharm.auth.gr (E.P.); thalialiargkova@yahoo.gr (T.L.); hadjipav@pharm.auth.gr (D.H.-L.)

[4] Department of Food Science and Technology, University of West Attica, Ag. Spyridonos, 12243 Egaleo, Greece; pzoump@uniwa.gr (P.Z.)

* Correspondence: adetsi@chemeng.ntua.gr; Tel.: +30-210-7724126

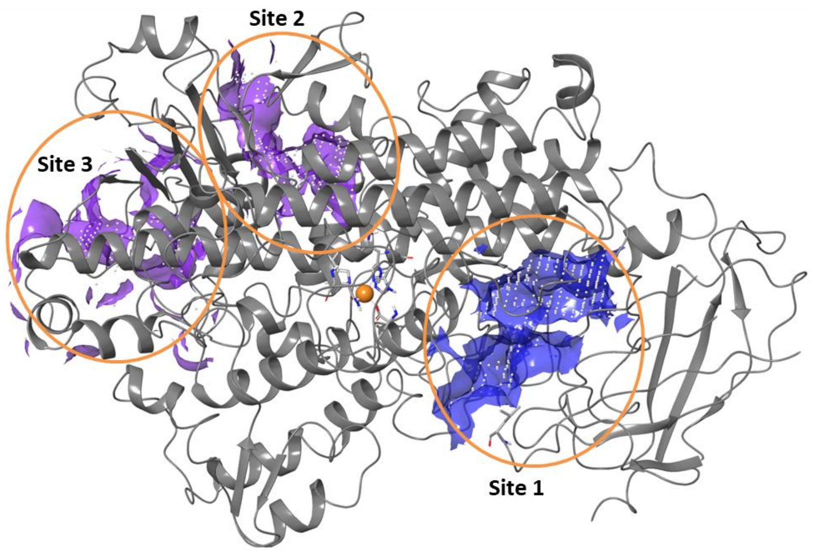

Figure S1: Predicted binding sites of Soybean Lipoxygenase seed isoform 1 (LOX-1).

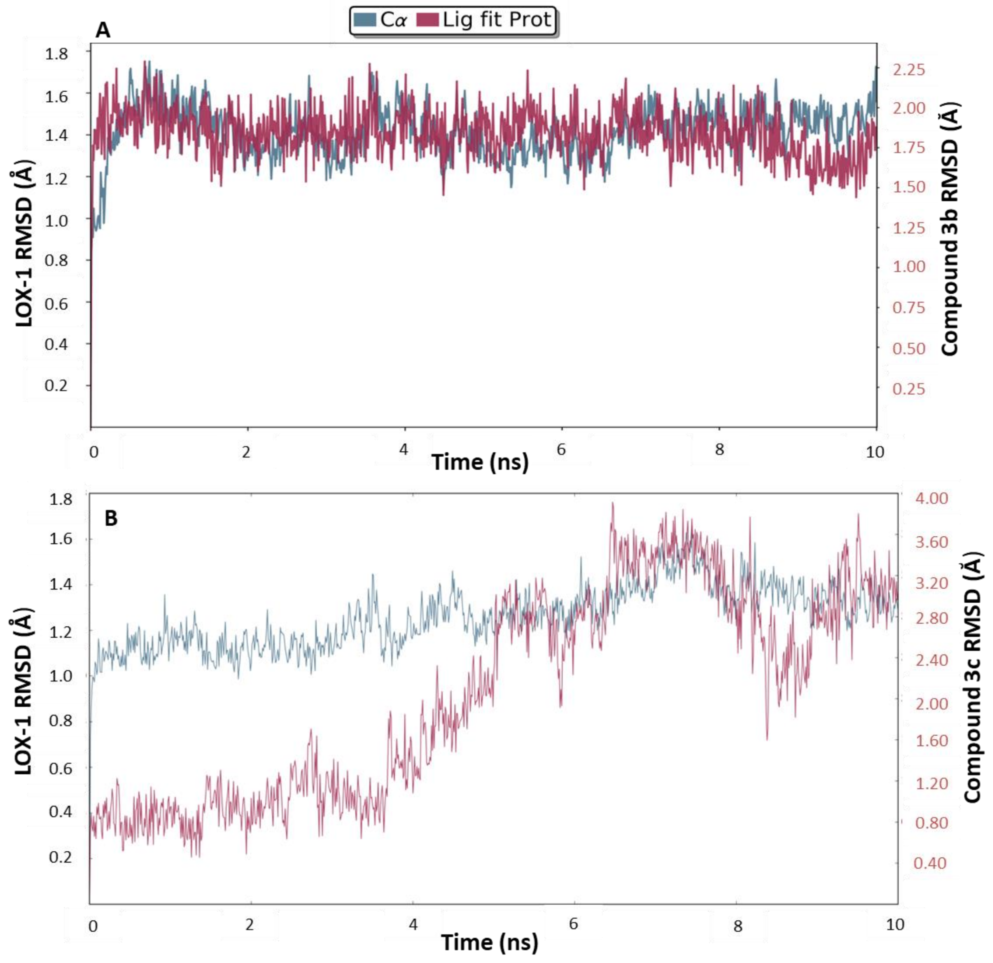

Figure S2: RMSD calculations for (A) all Ca enzyme atoms (blue) in LOX-1-Compound **3b** complex and for all atoms of compound **3b** (red), (B)) all C_a enzyme atoms (blue) in LOX-1-Compound **3c** complex and for all atoms of compound **3c** (red).

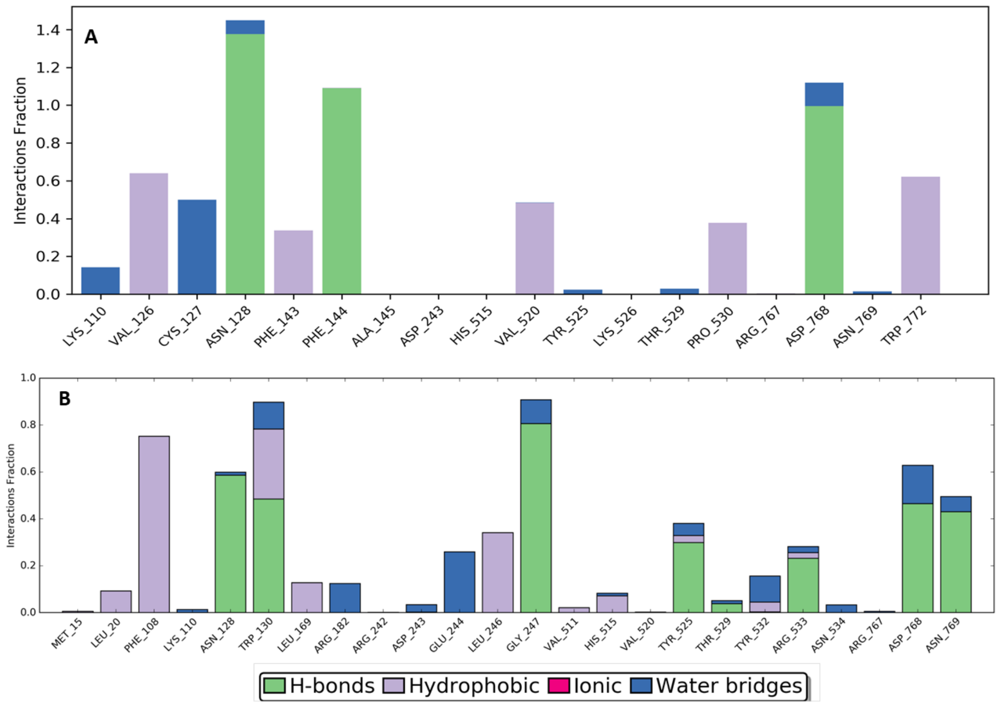

Figure S3: Categorization of (A) LOX-1-Compound **3b** and (B) LOX-1-Compound **3c** interactions, during the molecular dynamics simulation (10 ns).

^{1}H NMR spectra of all the synthesized compounds and ^{13}C NMR spectra of the novel synthesized compounds

^{1}H NMR of compound **3a**

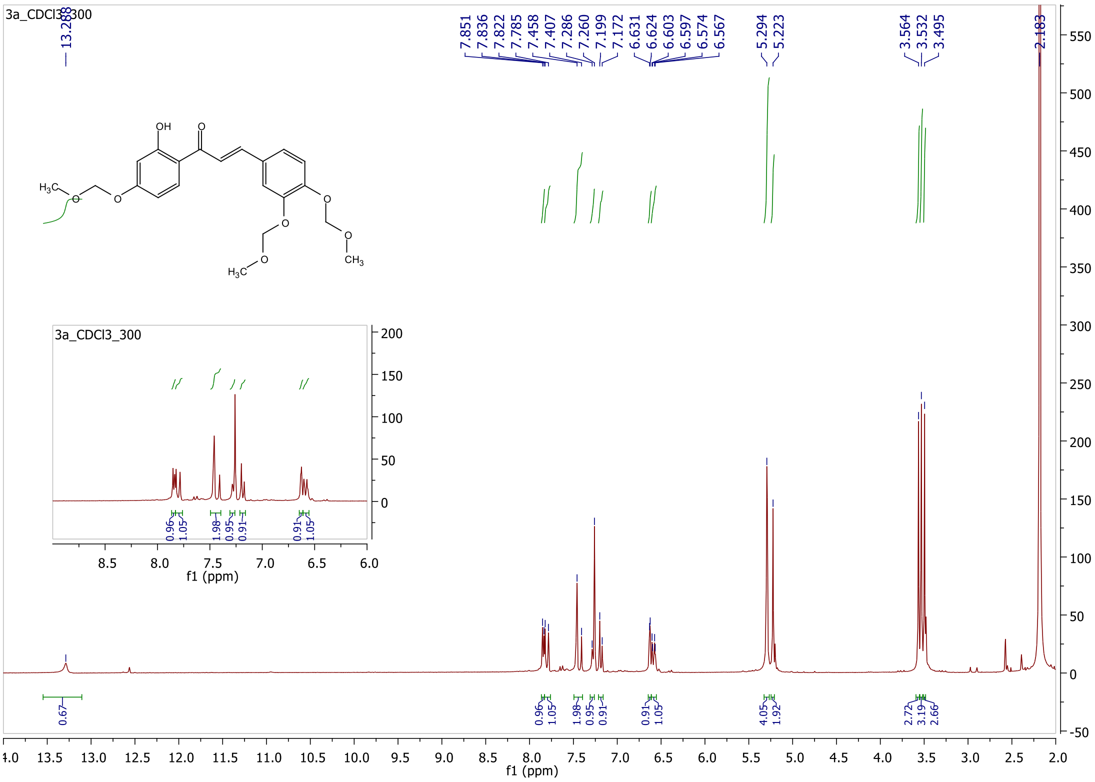

^{1}H NMR of compound **3b**

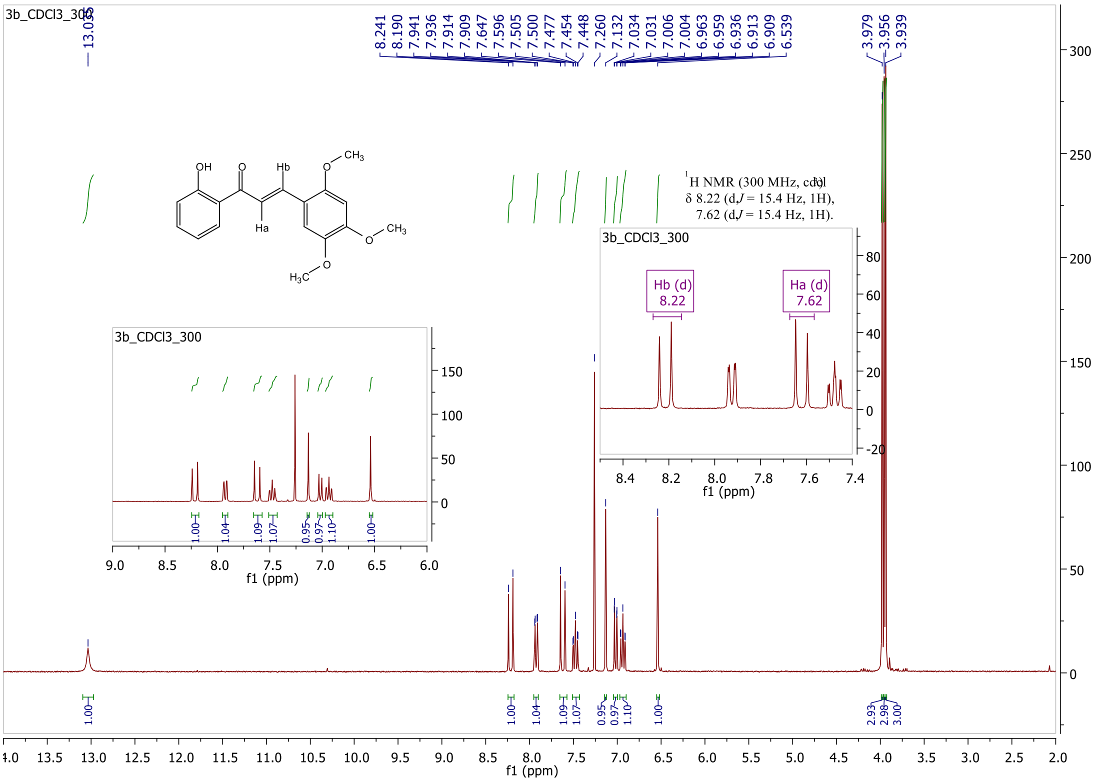

^{1}H NMR of compound **3c**

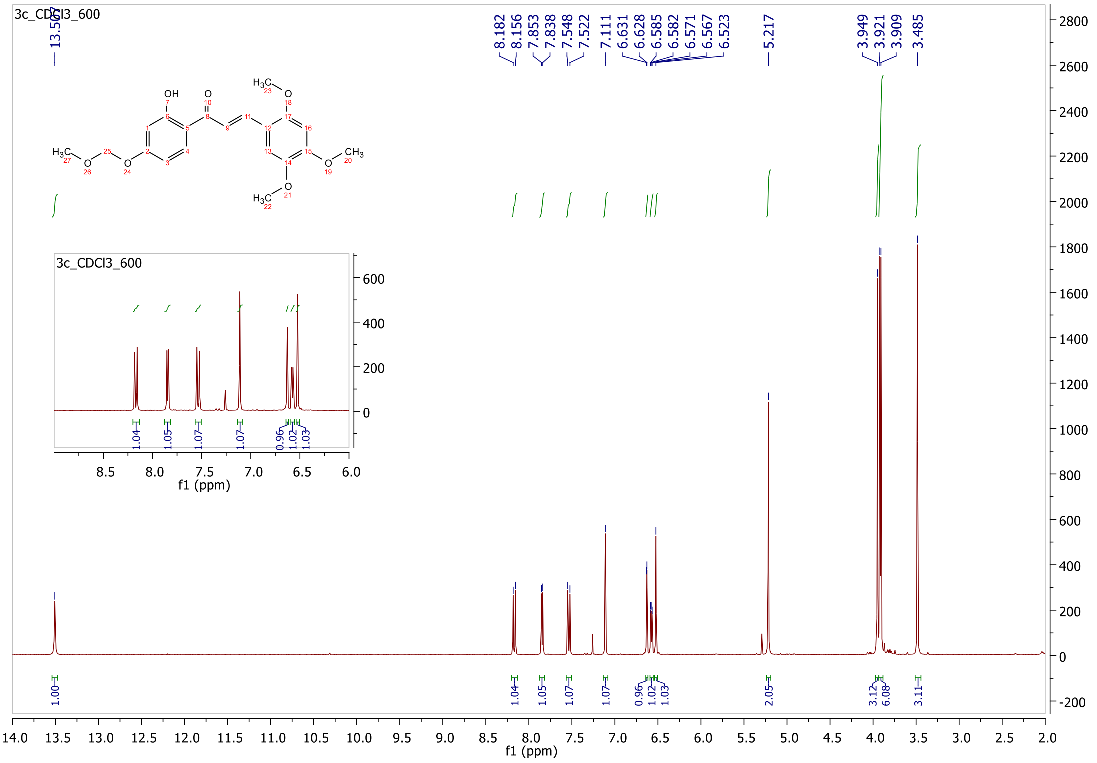

^{13}C NMR of compound **3c**

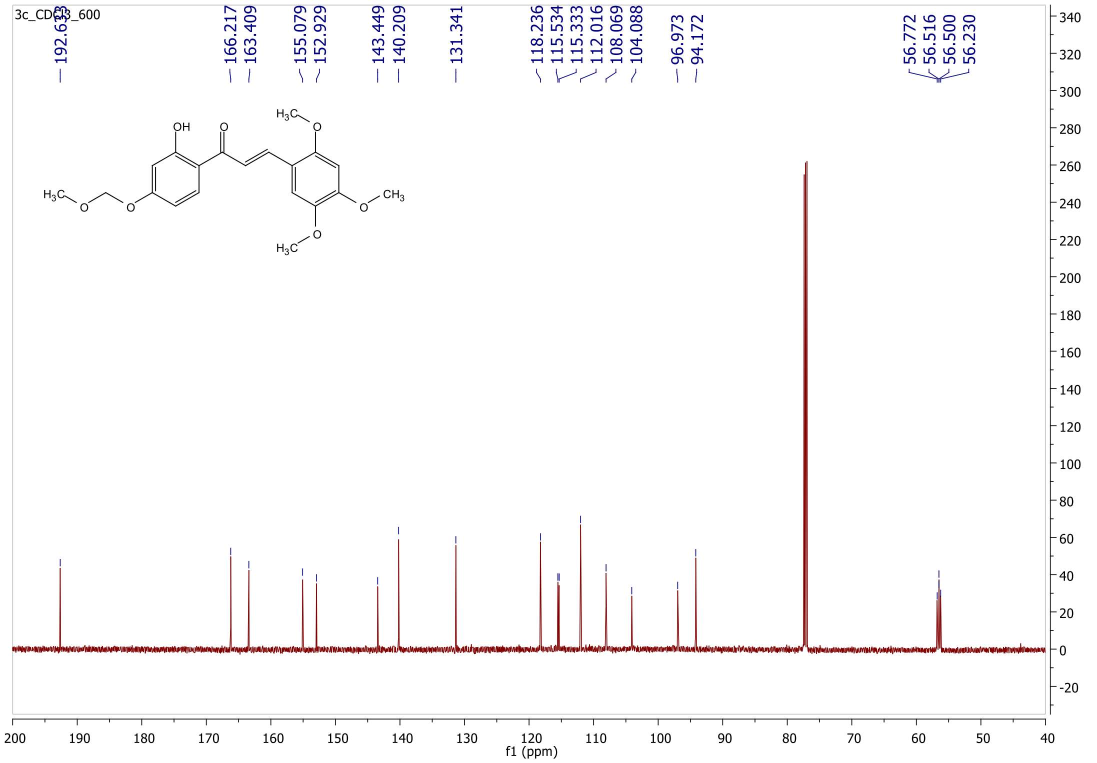

^{1}H NMR of compound **3d**

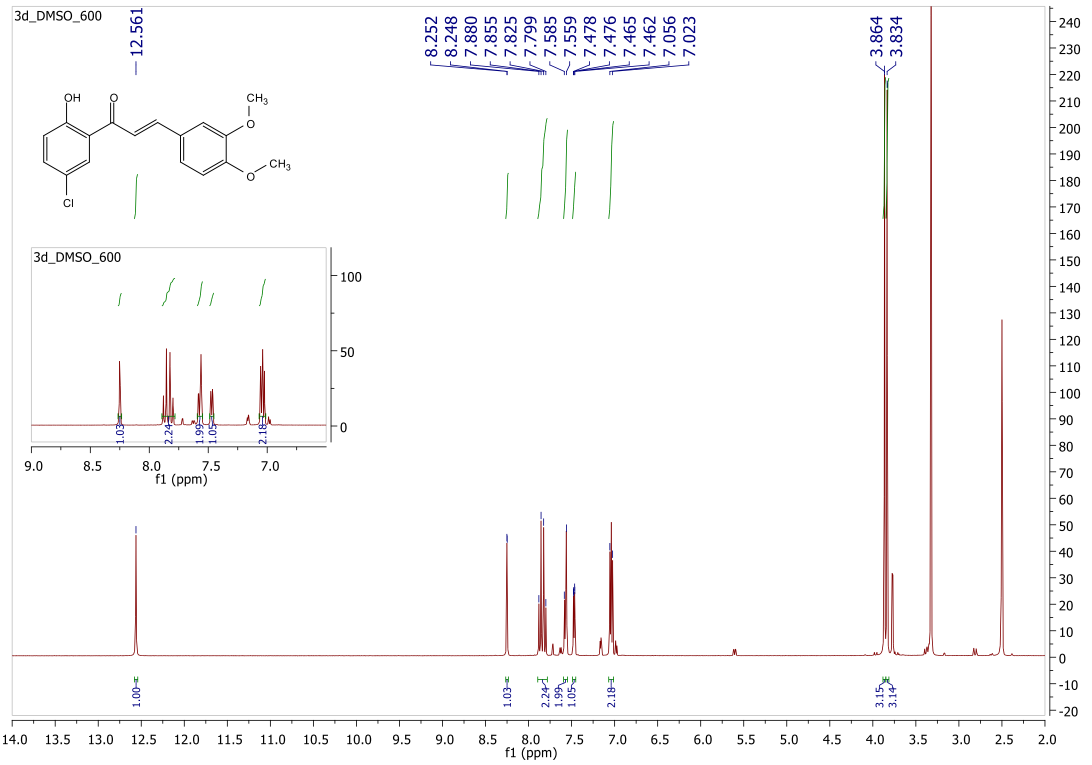

^{1}H NMR of compound **3e**

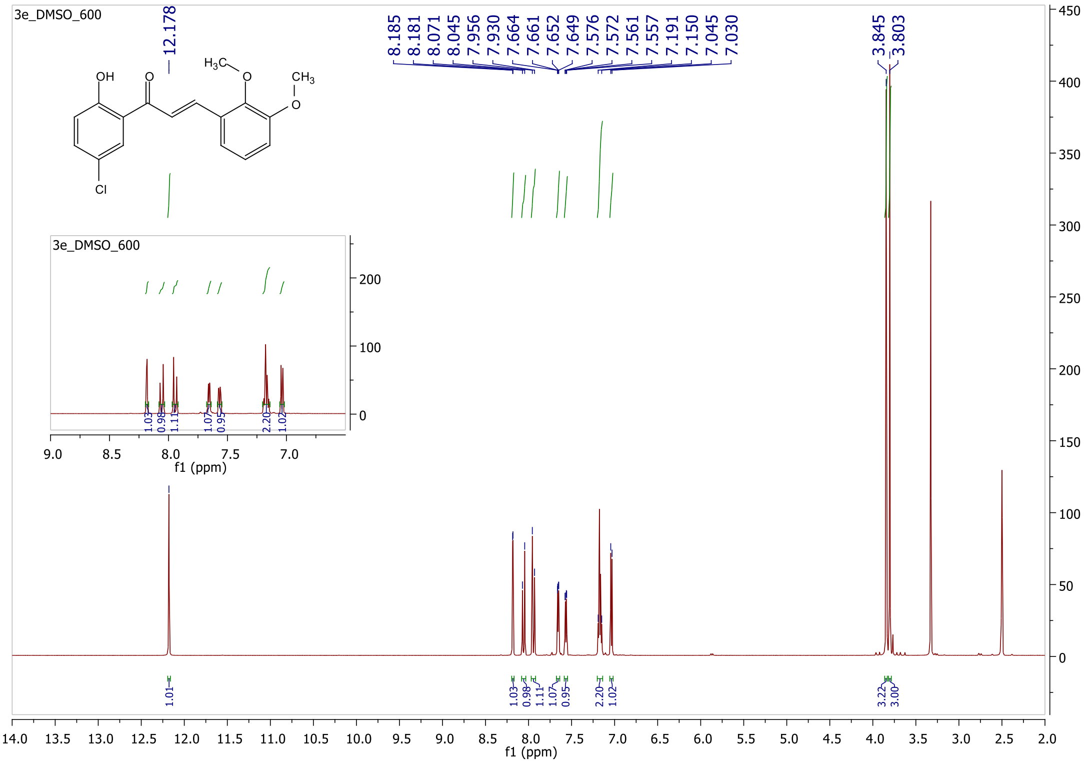

^{1}H NMR of compound **3f**

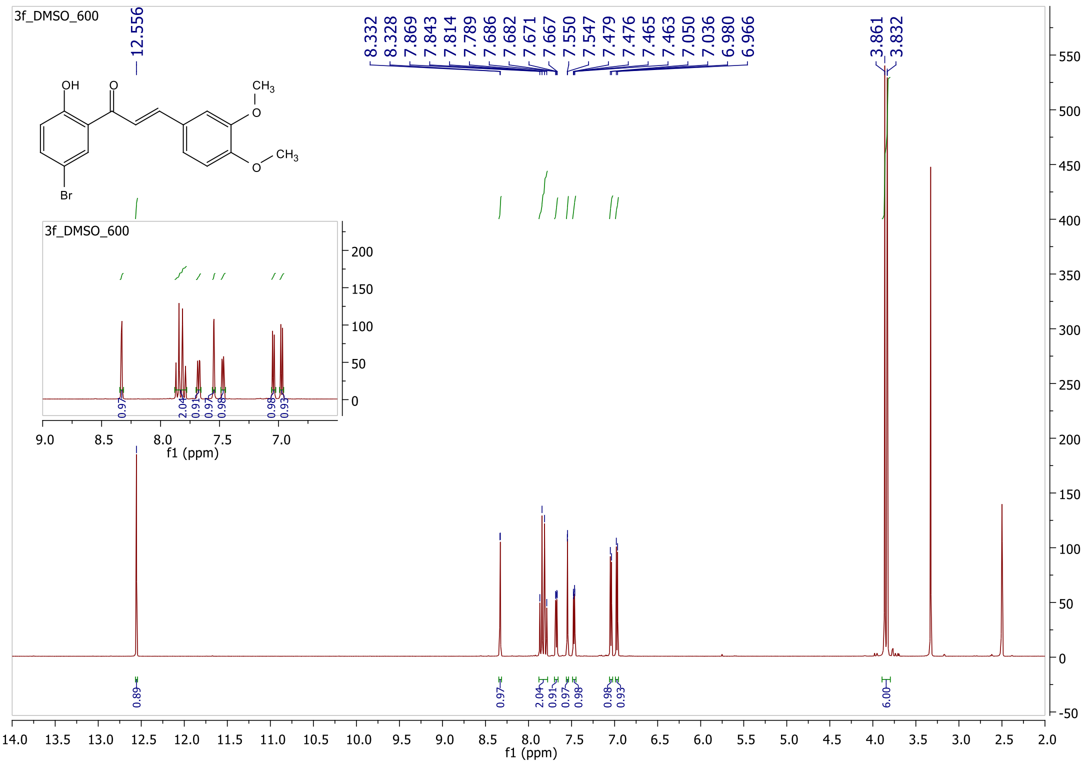

^{1}H NMR of compound **3g**

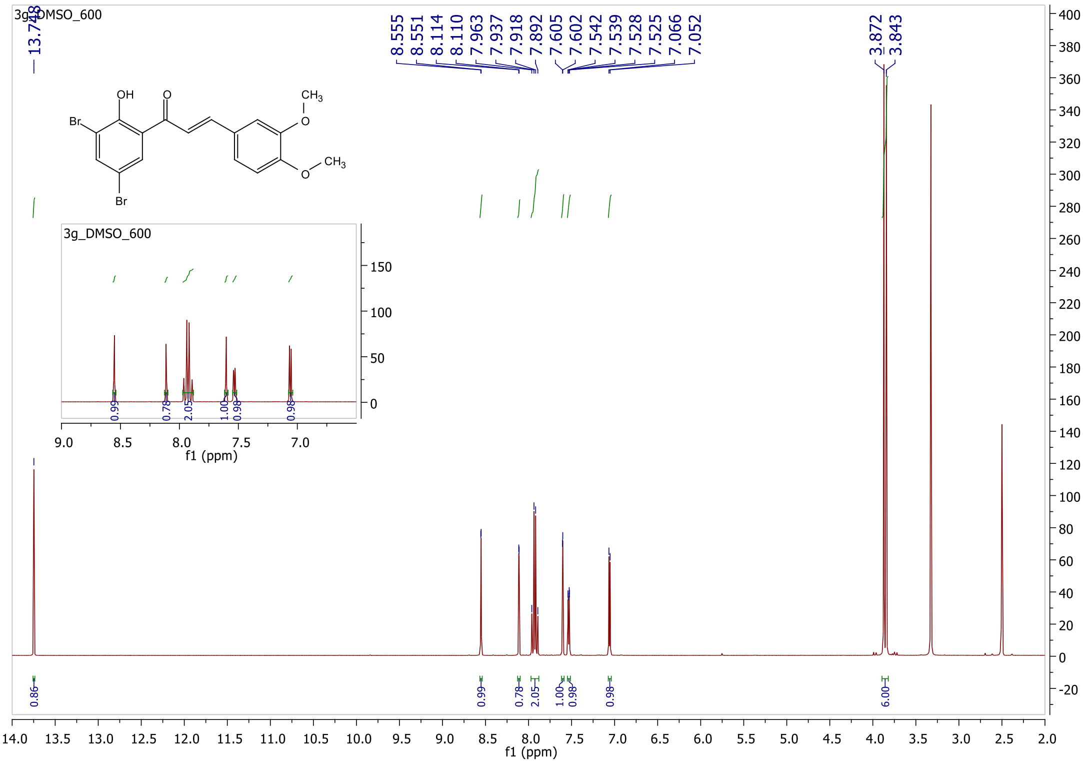

^{1}H NMR of compound **3h**

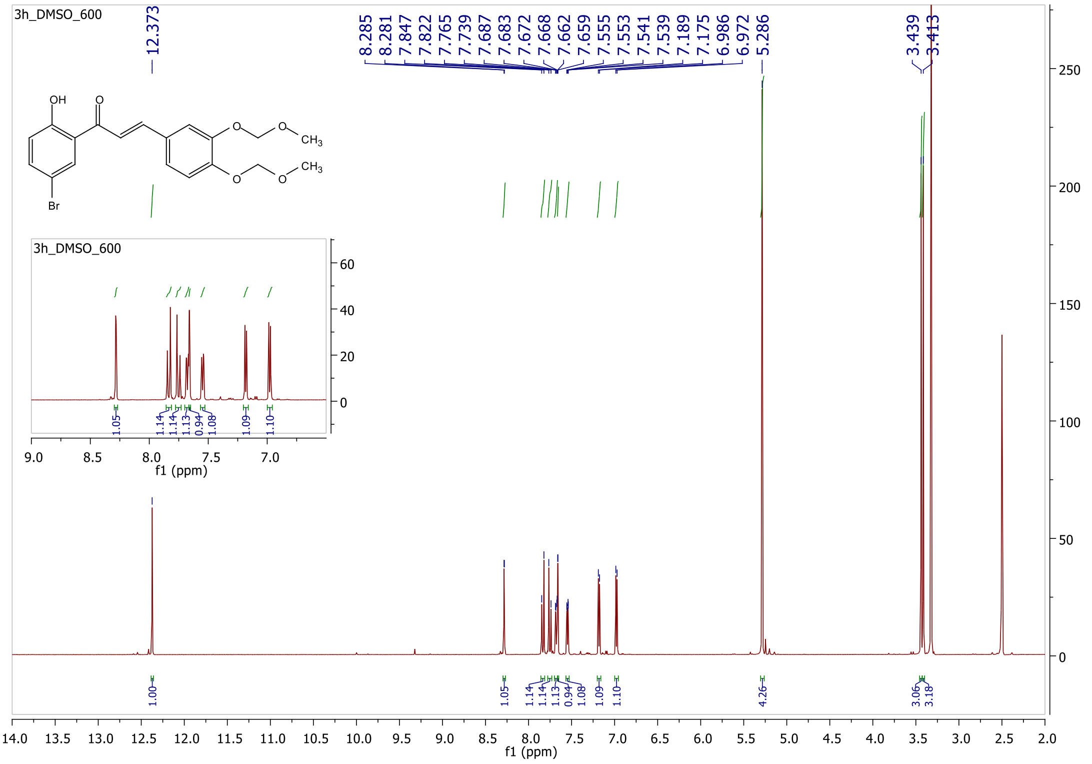

^{13}C NMR of compound **3h**

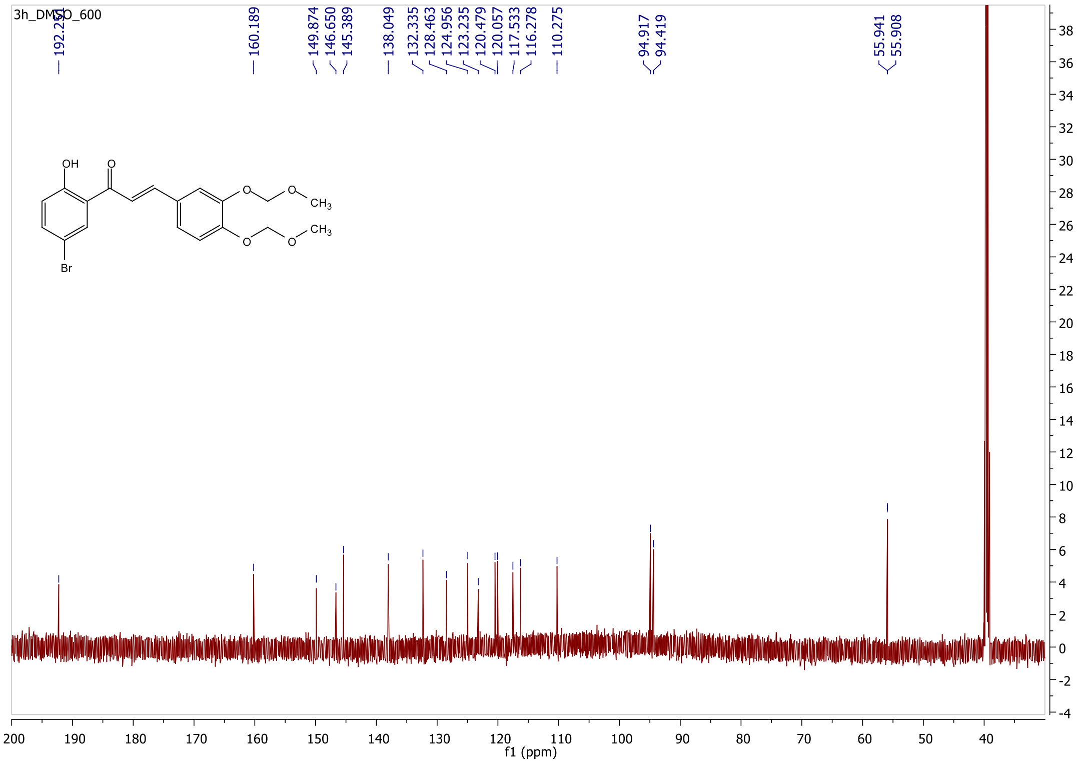

^1H NMR of compound **3i**

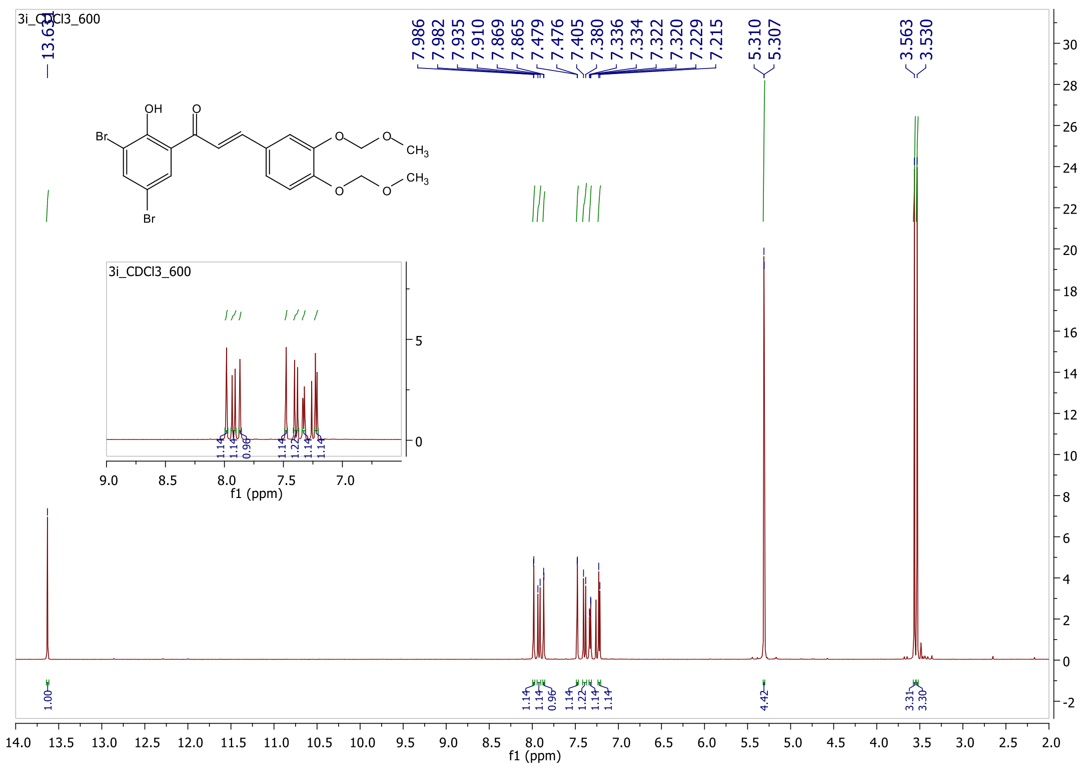

^1H NMR of compound **3j**

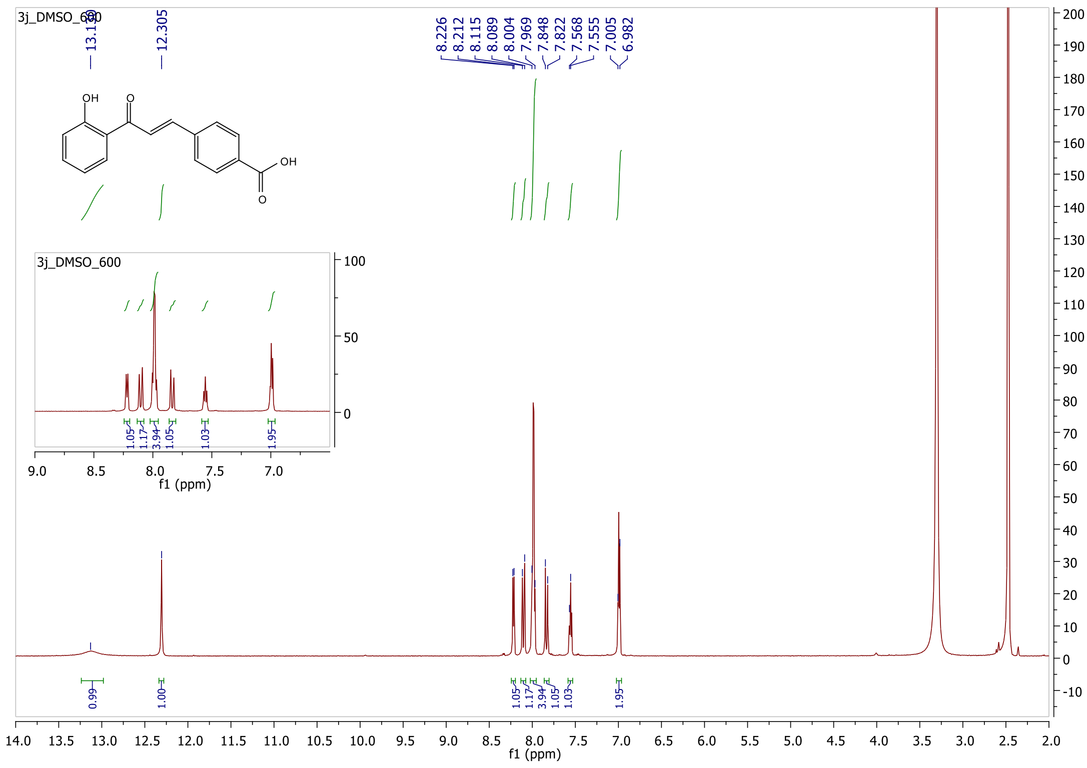

^{1}H NMR of compound **3k**

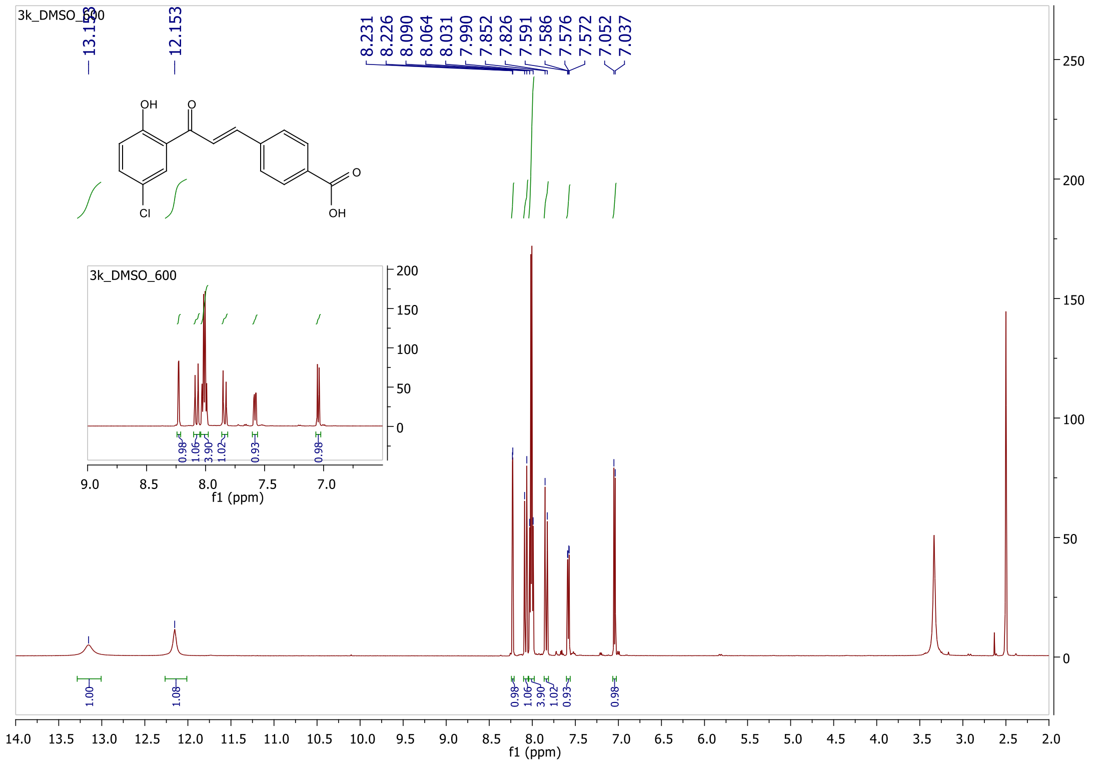

^{1}H NMR of compound **3l**

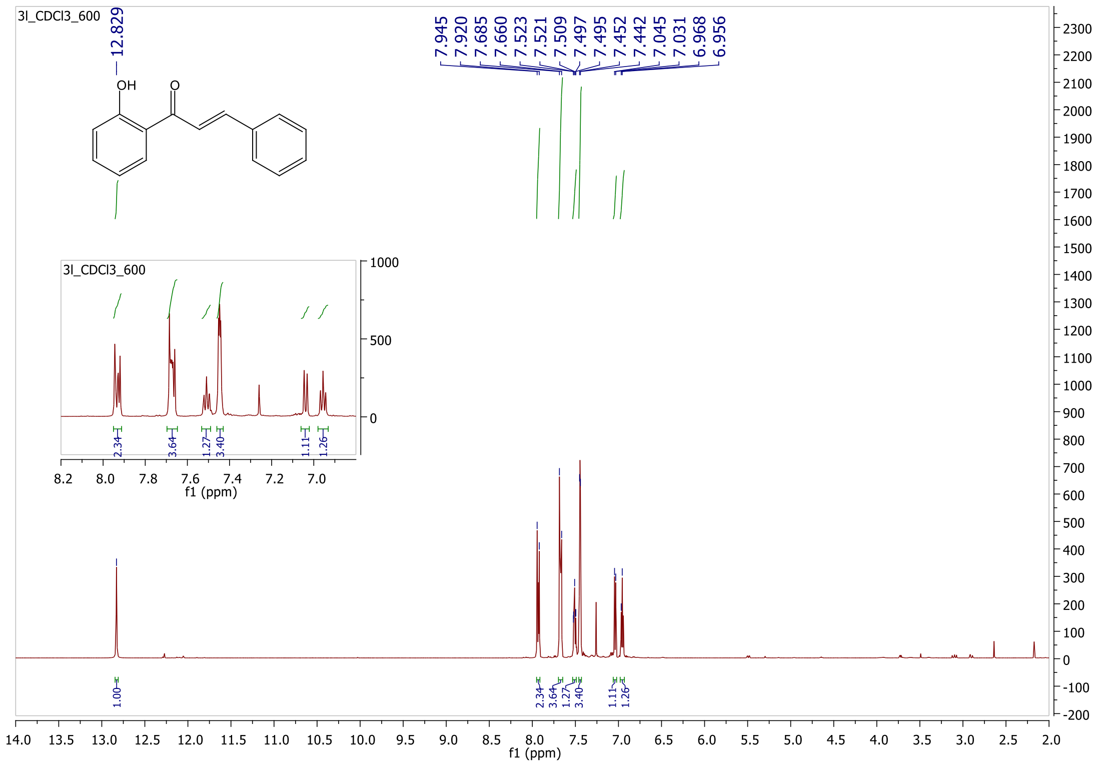

^{1}H NMR of compound **4a**

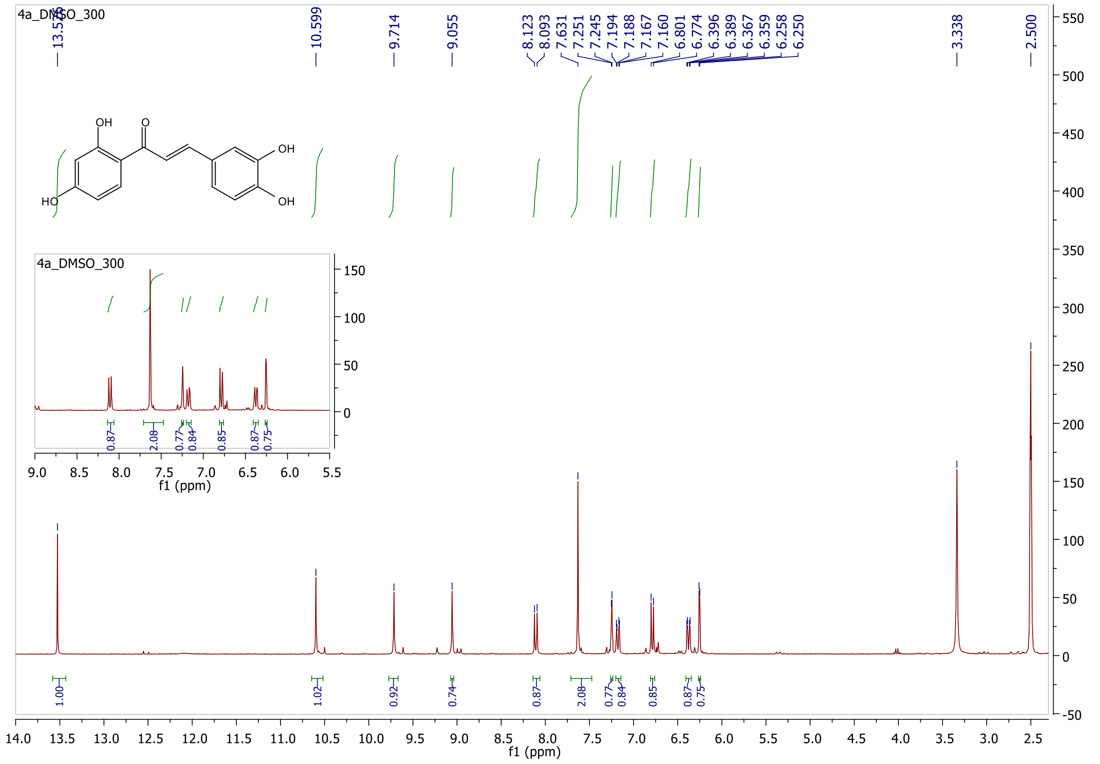

^{1}H NMR of compound **4b**

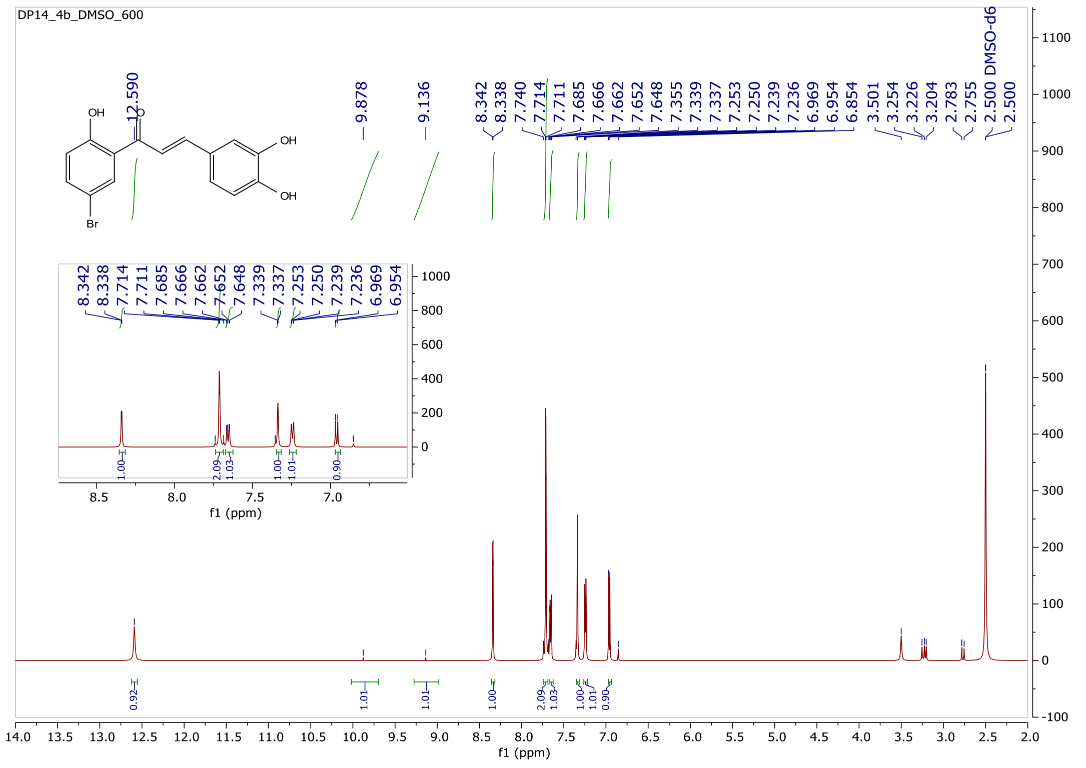

^{13}C NMR of compound **4b**

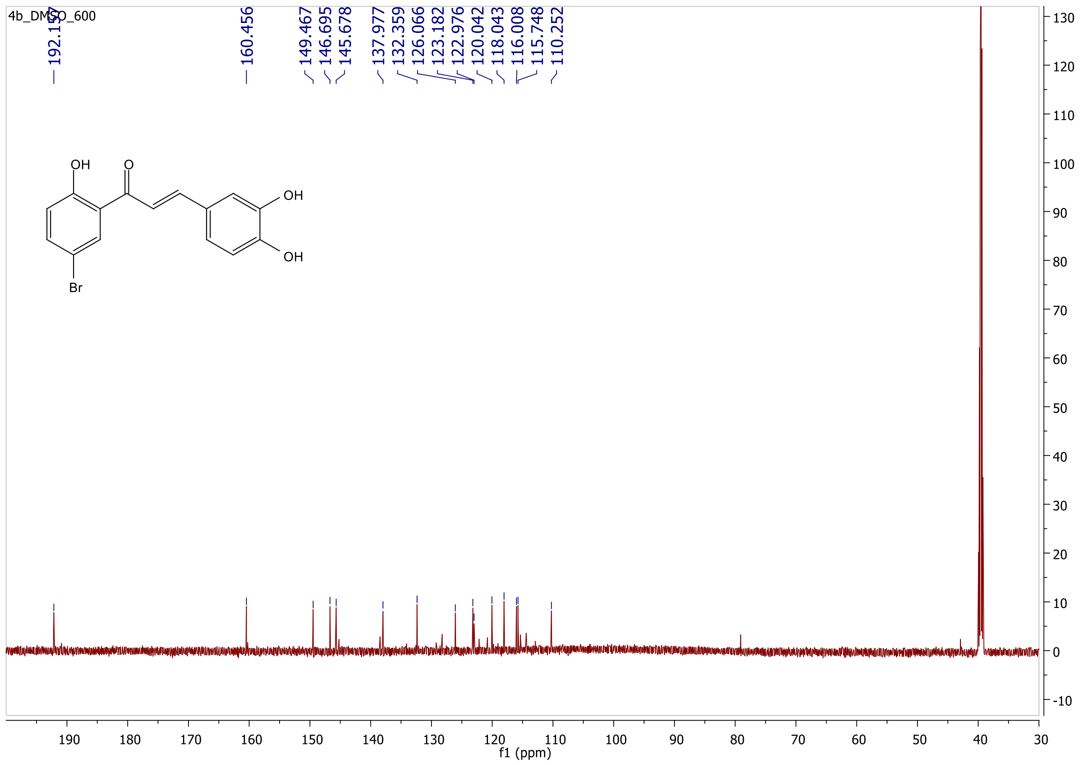

^{1}H NMR of compound **5**

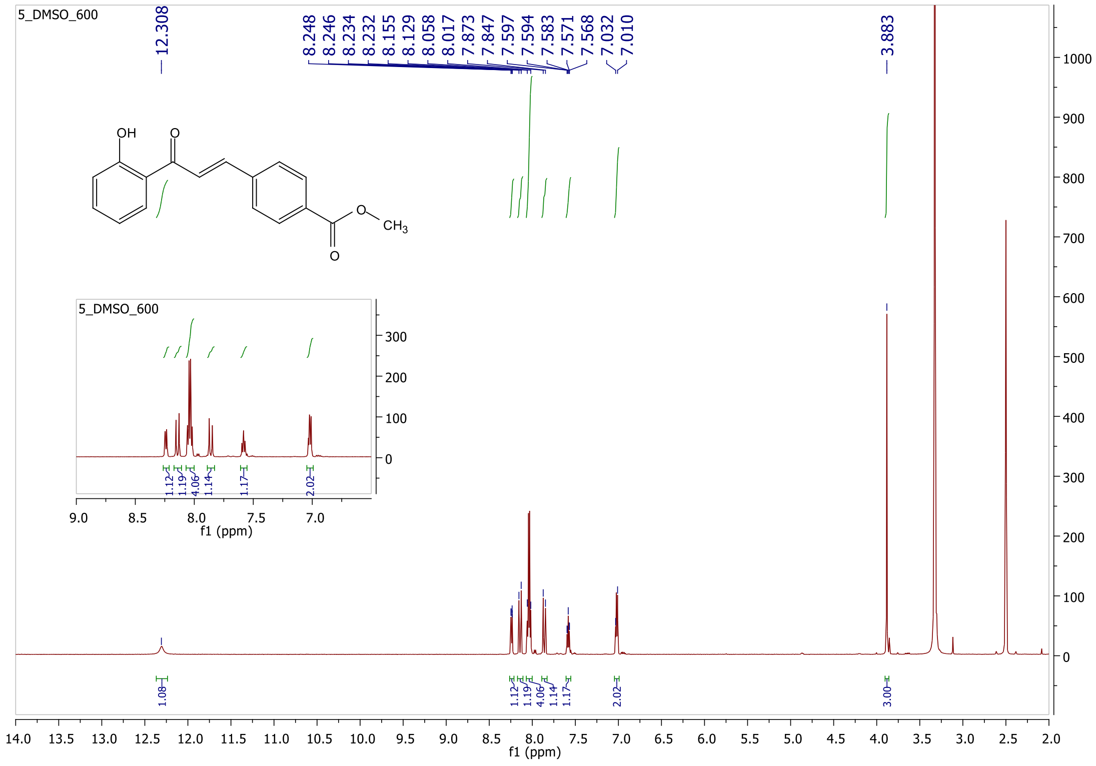

^{13}C NMR of compound **5**

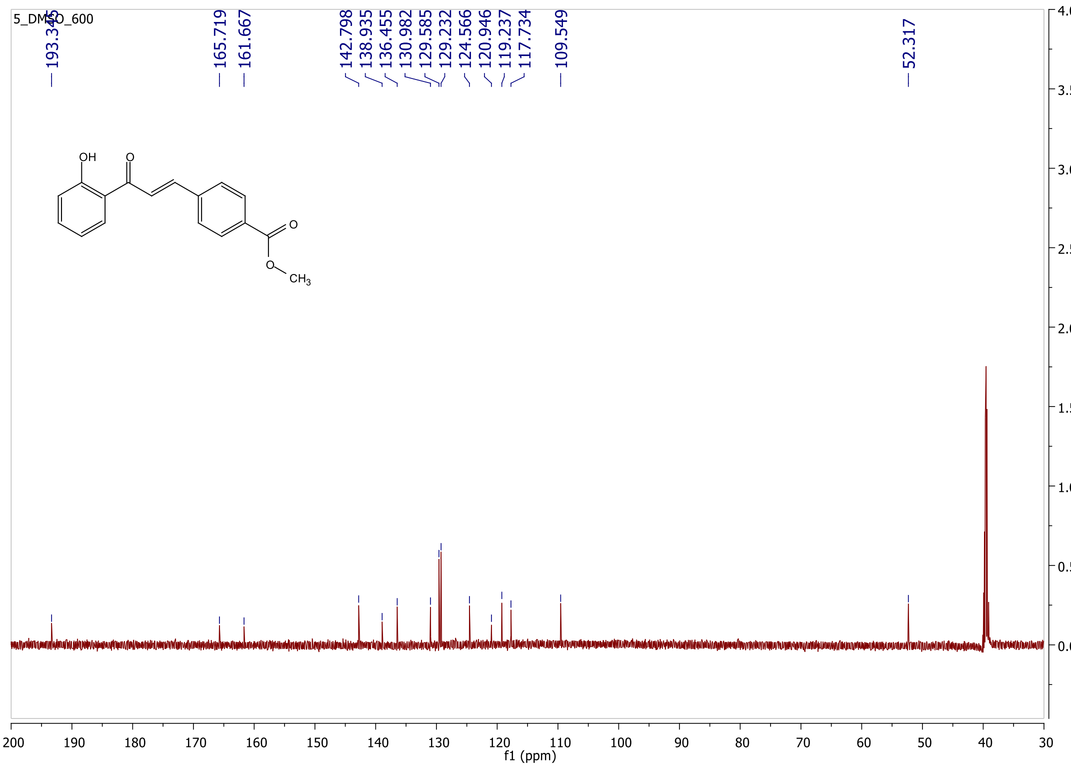

^{1}H NMR of compound **6a**

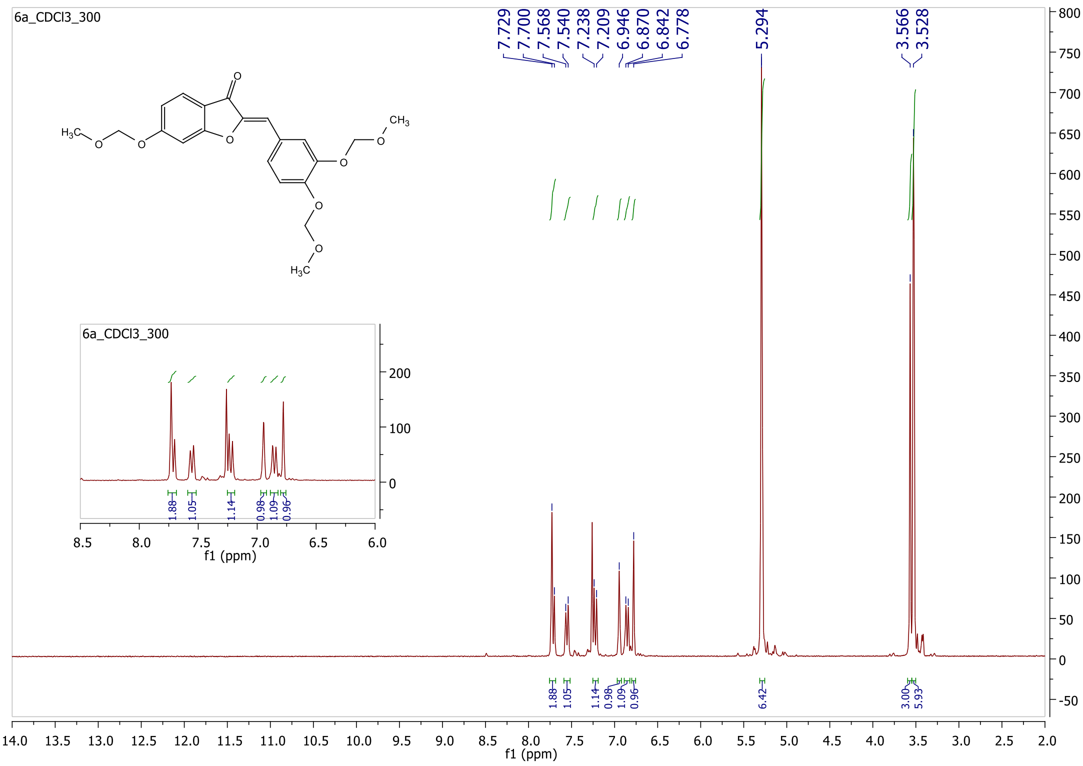

^{13}C NMR of compound **6a**

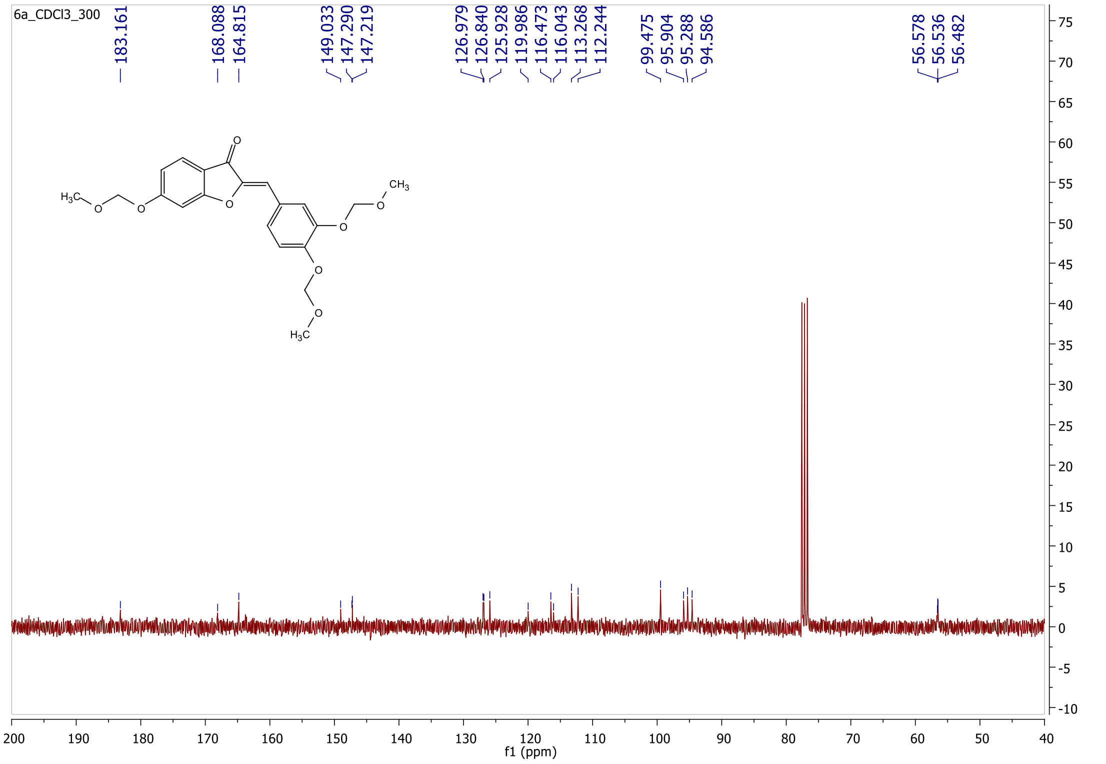

^{1}H NMR of compound **6b**

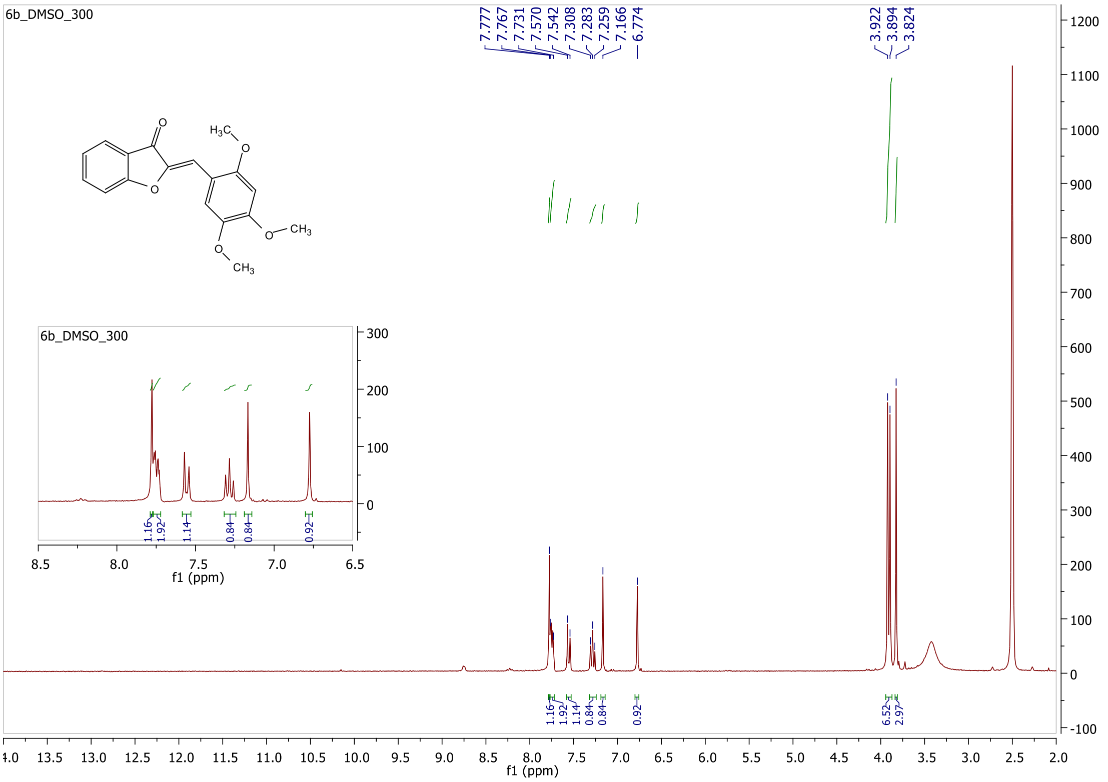

^{1}H NMR of compound **7a**

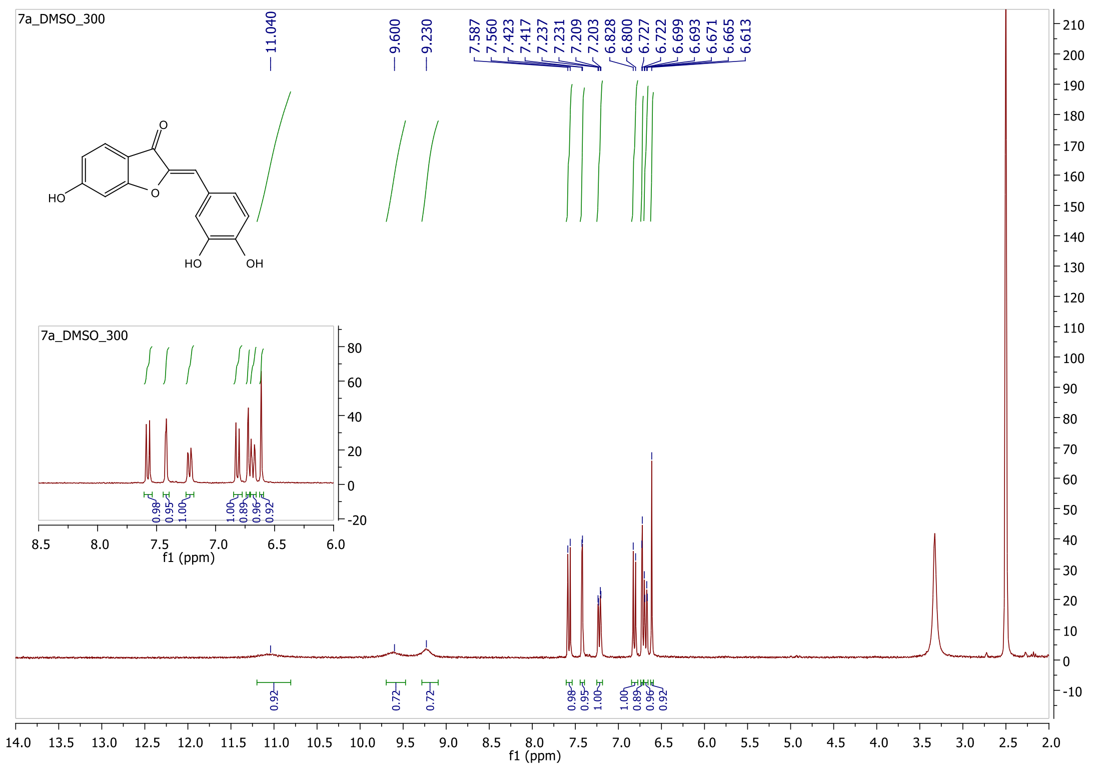

^{1}H NMR of compound **7b**

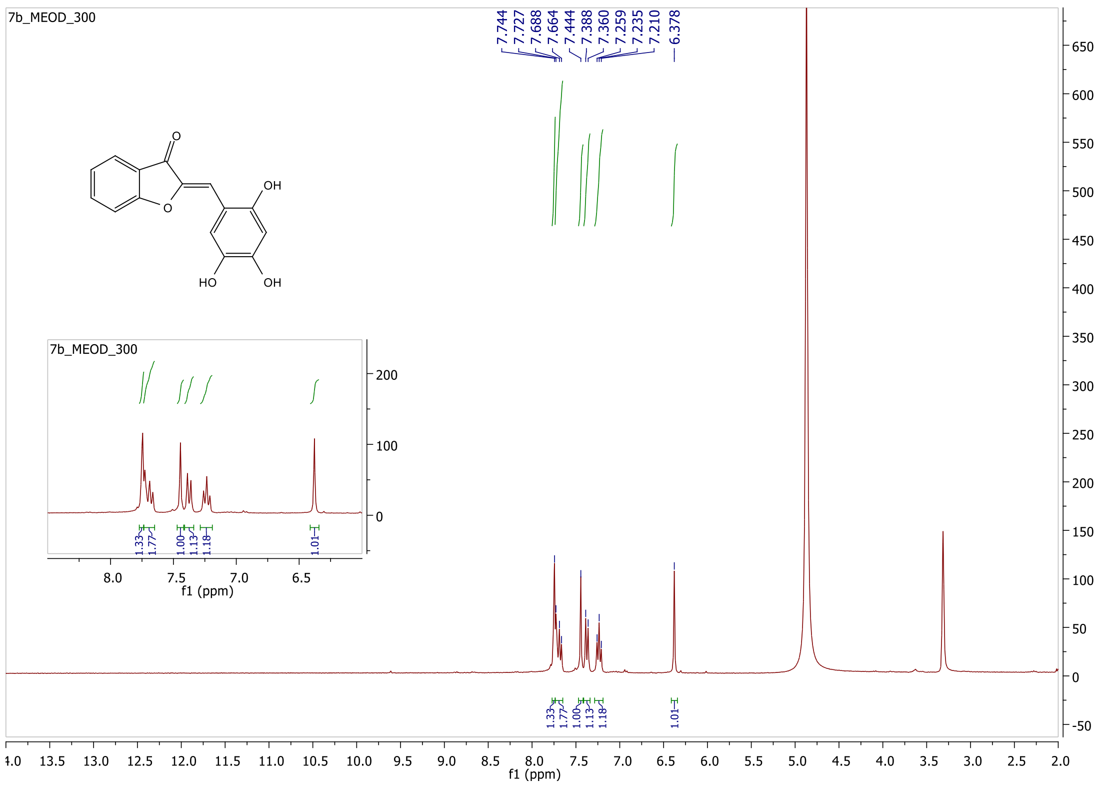

Supplement: Supplementary file 1 [file molecules-26-02777-s001.zip › molecules-1104228-supplementary.pdf]
